# Remodeling of the Tumor Microenvironment Through PAK4 Inhibition Sensitizes Tumors to Immune Checkpoint Blockade

**DOI:** 10.1158/2767-9764.CRC-21-0133

**Published:** 2022-10-19

**Authors:** Gabriel Abril-Rodriguez, Davis Y. Torrejon, Daniel Karin, Katie M. Campbell, Egmidio Medina, Justin D. Saco, Mildred Galvez, Ameya S. Champhekar, Ivan Perez-Garcilazo, Ignacio Baselga-Carretero, Jas Singh, Begoña Comin-Anduix, Cristina Puig-Saus, Antoni Ribas

**Affiliations:** 1 Department of Medicine, Division of Hematology-Oncology, University of California, Los Angeles (UCLA), Los Angeles, California.; 2 Department of Molecular and Medical Pharmacology, UCLA, Los Angeles, California.; 3 Arcus Biosciences, Inc., Hayward, California.; 4 Department of Surgery, Division of Surgical Oncology, UCLA, Los Angeles, California.; 5 Jonsson Comprehensive Cancer Center, Los Angeles, California.; 6 Parker Institute for Cancer Immunotherapy, San Francisco, California.

## Abstract

PAK4 inhibition can sensitize tumors to immune checkpoint blockade (ICB) therapy; however, the underlying mechanisms remain unclear. We report that PAK4 inhibition reverses immune cell exclusion by increasing the infiltration of CD8 T cells and CD103^+^ dendritic cells (DC), a specific type of DCs that excel at cross-presenting tumor antigens and constitute a source of CXCL10. Interestingly, in melanoma clinical datasets, *PAK4* expression levels negatively correlate with the presence of *CCL21*, the ligand for CCR7 expressed in CD103^+^ DCs. Furthermore, we extensively characterized the transcriptome of PAK4 knockout (KO) tumors, *in vitro* and *in vivo*, and established the importance of PAK4 expression in the regulation of the extracellular matrix, which can facilitate immune cell infiltration. Comparison between PAK4 wild type and KO anti-PD-1 treated tumors revealed how PAK4 deletion sensitizes tumors to ICB from a transcriptomic perspective. In addition, we validated genetically and pharmacologically that inhibition of PAK4 kinase activity is sufficient to improve antitumor efficacy of anti-PD-1 blockade in multiple melanoma mouse models. Therefore, this study provides novel insights into the mechanism of action of PAK4 inhibition and provides the foundation for a new treatment strategy that aims to overcome resistance to PD-1 blockade by combining anti-PD-1 with a small-molecule PAK4 kinase inhibitor.

**Significance:**

Our findings provide new insights into PAK4 inhibition mechanism of action as well as the scientific foundation for specifically blocking PAK4 kinase activity using a novel and specific PAK4 kinase inhibitor to overcome resistance to PD-1 blockade.

## Introduction

Cancer immunotherapy has changed the treatment landscape of multiple tumor types, including advanced melanoma (1). PD-1/PD-L1 blockade therapy works by releasing the brakes on the immune system and allowing the preexisting antitumor response to resume and eradicate cancer cells (2). Despite the unprecedented clinical success, with the approval by the FDA of multiple mAbs blocking the PD-1/PD-L1 axis, the majority of patients with cancer do not respond to treatment or relapse shortly after (3, 4). Lack of tumor infiltration by immune cells, which results in low IFNγ signature, constitutes one of the main mechanisms of resistance to ICB therapies (2, 5–7), and tumor-intrinsic oncogenic signaling pathways could drive immune cell exclusion from the tumor microenvironment (8–10). Among the different pathways, WNT/β-catenin signaling has been associated with poor immune cell infiltration and resistance to anti-PD-1 blockade therapy in melanoma and other tumor types (11–13). However, there is a paucity of targets that could potentially be inhibited to reverse immune cell exclusion and overcome resistance to PD-1 blockade therapies.

PAK4 is a member of the group II p21-activated kinases (PAK) family and functions as a central player in the reorganization of the cytoskeleton (14). PAK4 is also involved in several other cellular functions including cell survival and proliferation (15–17), but it is best known for its role in controlling cellular morphology, cell adhesion, and cell migration (18–20). Of note, *PAK4* overexpression is associated with tumorigenesis in several tumor types including breast, pancreatic, bladder, ovarian cancer, and melanoma (21–25), and constitutes a potential target for cancer treatment. We have recently shown that in melanoma, *PAK4* overexpression is associated with lack of immune cell infiltration and resistance to PD-1 blockade immunotherapy, with its inhibition resulting in increased immune cell infiltration leading to overcoming resistance to anti-PD-1 therapy (13). In addition, it has been shown that deletion of the *PAK4* gene in endothelial cells remodels the vascular microenvironment leading to increased T-cell infiltration and improved responses to chimeric antigen receptor (CAR)-T immunotherapy in glioblastoma (26). However, the specific mechanisms underlying the improvement of effectiveness of ICB remain largely unknown.

Here, we show how PAK4 inhibition increases not only T-cell infiltration, but also CD103^+^ dendritic cell (DC) infiltration, an important subset of dendritic cells that excel at cross-presenting tumor antigens and priming T cells. We also describe how lack of *PAK4* expression alters the tumor microenvironment and sensitizes murine melanoma to anti-PD-1 therapy from a transcriptomic perspective. Finally, we demonstrate that the kinase activity of PAK4 is responsible for the improved response and show that pharmacologic inhibition of PAK4 activity with a specific PAK4 kinase inhibitor improves the efficacy of anti-PD-1 immunotherapy. Taken together, these findings provide the rationale for a novel treatment strategy to overcome PD-1 blockade resistance by administering a combination of anti-PD-1 in combination with a specific PAK4 kinase inhibitor.

## Materials and Methods

### Cell Lines and PAK4 Kinase Dead Generation

Murine B16 cells were obtained from the ATCC and YUMM2.1 cells from Bosenberg's lab. Cell lines were authenticated with IDEXX BioAnalytics. YUMM2.1, B16 cells and cells derived from this parental cell line [B16 PAK4 knockout (KO) and B16 PAK4 kinase dead (KD)] were maintained in DMEM, supplemented with 10% FBS, 100 units/mL penicillin, and 100 μg/mL streptomycin at 37°C in a humidified atmosphere of 5% CO_2_. All cells were maintained and confirmed *Mycoplasma* negative using MycoAlert *Mycoplasma* Detection Kit (Lonza). An early passage stock for each cell line was thawed before experiments and only used for *in vitro* and *in vivo* assays within passages 3 and 13 after thawing. To generate a B16 PAK4 KD cell line, we took advantage of the previously generated lentiviral vector with the PAK4 open reading frame (ORF) and performed site directed mutagenesis with the following primers containing the mutation to change AAG codon to ATG (K352M): 5-GGCAAACTGGTGGCCGTCATGAAGATGGACTTGCGCAAGC-3 and 5-GCTTGCGCAAGTCCATCTTCATGACGGCCACCAGTTTGCC-3. After cloning and vector amplification on Stbl3, 293T cells were used for lentiviral particle generation and B16 PAK4 KO cells were transduced at 20% confluency. A total of 24 hours after transduction, media was changed and cells were expanded and sorted on the basis of Thy1.1 expression. PAK4 expression and loss of kinase activity was validated by Western blot and Topflash assay.

### Mouse Model Studies

All mouse studies were approved by the University of California, Los Angeles (UCLA) Animal Research Committee (protocol #2004-159-23). C57BL/6 mice were bred and kept under defined-flora pathogen-free conditions at the Association for Assessment and Accreditation of Laboratory Animal Care–approved animal facility of the Division of Experimental Radiation Oncology, UCLA. To study the *in vivo* effects of genetically blocking the kinase activity of PAK4 effect on PD-1 blockade efficacy, we subcutaneously injected 0.3 × 10^6^ B16 PAK4 KD and B16 PAK4 rescue into the flanks of C57BL/6 syngeneic mice. A total of 96 hours after tumor injection, mice were randomly assigned into the different groups. Anti PD-1 (catalog no. BE0146, clone RMP1-14, BioXCell) treatment was injected intraperitoneally three times per week at 300 μg per dose. We followed the same protocol to test the impact of pharmacologic inhibition of PAK4 kinase activity. For these experiments, we also used the YUMM2.1 melanoma cell line, which was injected subcutaneously (1 × 10^6^ cells) into the flanks of C57BL/6 syngeneic mice. We established four treatment groups of B16 wild type (WT) CRISPR control (CC), or YUMM2.1 melanoma cells: (i) vehicle, (ii) anti-PD-1, (iii) PAK4 inhibitor (A0317859), and (iv) combination. The PAK4 inhibitor was administered by oral gavage at 300 mg/kg daily and anti-PD-1 was injected intraperitoneally three times per week at 300 μg per dose. In all *in vivo* studies, tumor progression was monitored three times per week by measuring two perpendicular dimensions with a caliper.

### Flow Cytometry

To characterize and quantify the DC population, we collected mouse tumor samples from B16 WT CC or PAK4 KO melanoma cells treated with anti-PD-1 or vehicle at two different timepoints, day 6 and day 10. As described previously (13), tumor samples were processed using the mouse tumor dissociation kit (Miltenyi Biotec) following manufacture's protocol. Samples were stained using the antibodies listed in Supplementary Table S1. Following staining, samples were analyzed using the Attune Flow Cytometer (Thermo Fisher Scientific) platform at the UCLA Flow Cytometry core. Samples were analyzed using FlowJo software (v10.4.2).

### RNA Sequencing Analysis

To study the transcriptomic differences between PAK4 KO and WT cells, we harvested and collected RNA from a total of 18 *in vitro* samples (12 KO and 6 WT), using the RNeasy mini kit (Qiagen). These samples also included some that have been previously stimulated with either TNF at 100 ng/mL for 6 hours (Peprotech), IFNγ at 100 UI/mL for 6 hours (Peprotech) or Wnt-3a at 200 ng/mL for 8 hours (R&D Systems). We also isolated RNA from *in vivo* B16 WT CC and KO tumors as described previously (13). Samples were sequenced using the Illumina NextSeq500 platform with a read length of 1 × 75 at the UCLA Technology Center for Genomics & Bioinformatics. Raw FASTQ files were aligned to the GRCh38 genome (human) and GRCm38 genome (mouse) using HISAT2 version 2.1.0 (27) using the default parameters and counted with HTseq version 0.6.1p1 (28). Differential gene expression was performed on the basis of the negative binomial distribution with the DESeq2 package using default settings. To perform principal component analyses (PCA) with the DESeq2 package (29), raw reads were previously normalized using the variance stabilizing transformation function. We also used gene set enrichment analysis (GSEA) analysis with the following gene sets: C2 Curated Gene Sets and C5 Gene Ontology Gene Sets (30) to identify which signaling pathways were enriched in each of the different groups. Boxplots for Figs. 2D and 4D were done using the FPKMs rather than the normalized counts.

### IHC Analysis

Tissues were fixed in 10% neutral buffered formalin, processed and embedded in paraffin, and sectioned at 4 μm thickness using standard histologic procedures. Slides were dewaxed using xylene and rehydrated with a graded series of ethanol using a DAKO Coverstainer (DAKO, Agilent Technologies). Antigen retrieval was performed in a high pH buffer using PT Link (DAKO, Agilent Technologies) at 95°C for 20 minutes. IHC was carried out using a DAKO Autostainer Link 48 platform (DAKO, Agilent Technologies). Briefly, slides were blocked for endogenous peroxidases and subsequently stained using the following primary antibodies: rabbit anti-mouse CD8 (D4W27 at 1:200), Rabbit anti-mouse CD45 (Cell Signaling Technology D3F8Q at 1:200), and Rabbit anti-mouse CD31 antibodies (Abcam EPR17259 at 1:500) in Da Vinci diluent (Biocare Medical). MACH2 Rabbit AP polymer (Biocare Medical) was used to detect primary antibodies, followed by detection using Enzo Red chromogen (Enzo Life Sciences). Slides were then counterstained with Tacha's Hematoxylin (Biocare Medical), dehydrated, and coverslipped using the DAKO Coverstainer. Once dried, slides were scanned using a 3D Histech Pannoramic MIDI II Scanner (3DHISTECH), then image/spatial analyses were performed using HALO software (Indica Labs).

### WNT Activity Assays

Protein levels and S675-phosphorylation of β-catenin were evaluated by Western blot analysis as described previously (13) using the following antibodies: β-catenin (catalog no. 9587) and phospho-β-catenin (S675; catalog no. 9567) from Cell Signaling Technology. Nuclear and cytoplasmic extractions were performed with NE-PERTM Nuclear and Cytoplasmic Extraction Reagents (Thermo Fisher Scientific) following the manufacture's protocol. Topflash WNT activity assay were performed as described previously (13) using pSV-β-galactosidase control vector (PR-E1081, Promega), pTopflash (Addgene, catalog no. 12456) and mouse recombinant Wnt-3a (R&D Systems). For luciferase activity detection, we used the Bright-Glo Luciferase Assay System (Promega, catalog no. PR-E2610) and the Beta-Glo Assay System (Promega, catalog no. PR-E4720).

### Protein-level Quantification

*CXCL10* expression was measured by RT-PCR following the manufacturer's protocol for the Power SYBR Green RNA-to-CT 1-Step Kit (Applied Biosystems) and using the following FW and RV primers: 5-AATCATCCCTGCGAGCCTAT-3 and 5-TTTTTGGCTAAACGCTTTCAT-3.

*CCL21* protein expression was measured with the mouse 6-Ckine (CCL21A) ELISA kit (Thermo Fisher Scientific) following the manufacturer's protocol.

Mouse MHC class I and II were analyzed by flow cytometry with the following antibodies: MHC class I (H-2Kb) mAb (AF6-88.5.5.3), APC, eBioscience and MCH Class II (I-A/I-E) mAb (M5/114.15.2), APC, eBioscience.

### Statistical Analysis

GraphPad Prism7 (GraphPad Software, Inc), R software (v3.5.1), and FlowJo Version 10.7.1 was used for graphic representation and statistical analysis. Comparisons of CD103^+^ DC were performed using an unpaired *t* test with Welch correction. As described previously (13), differential gene expression was performed using the R package DESeq2 in which *P* values were calculated using the negative binomial generalized linear model fitting and the Wald significance test. The adjusted *P* values (*q*) were obtained by applying the Benjamini–Hochberg method. For *in vivo* studies, statistical significance and correction for multiple comparisons was calculated using the Holm-Sidak method. For *in vivo* studies with YUMM2.1 cells, we also performed a linear mixed-effects model analysis for tumor volume ran with terms for group, time and group*time interaction with a random mouse effect. Slopes were estimated for each group and pairwise contrasts were estimated using the model. Models were run using SAS V9.4 (SAS Institute) and *P* values <0.05/6, or 0.008 Bonferroni-adjusted) were considered statistically significant. When comparing the expression of a gene or signature between only two groups, we used a two-tailed unpaired *t* test. If multiple groups were tested, then we used a one-way ANOVA and corrected for multiple comparisons using statistical hypothesis testing with the Tukey test. Differences were considered statistically significant if *P* < 0.05.

### Data Availability

The data generated in this study are available within the article and its Supplementary Data. The sequence data generated in this study has been submitted to the NCBI BioProject database PRJNA876309 (https://www.ncbi.nlm.nih.gov/bioproject/).

## Results

### PAK4 Inhibition Increases CD103^+^ DC Infiltration and its Expression is Associated with *CCL21* Levels in Biopsies of Patients with Melanoma

**FIGURE 1:**
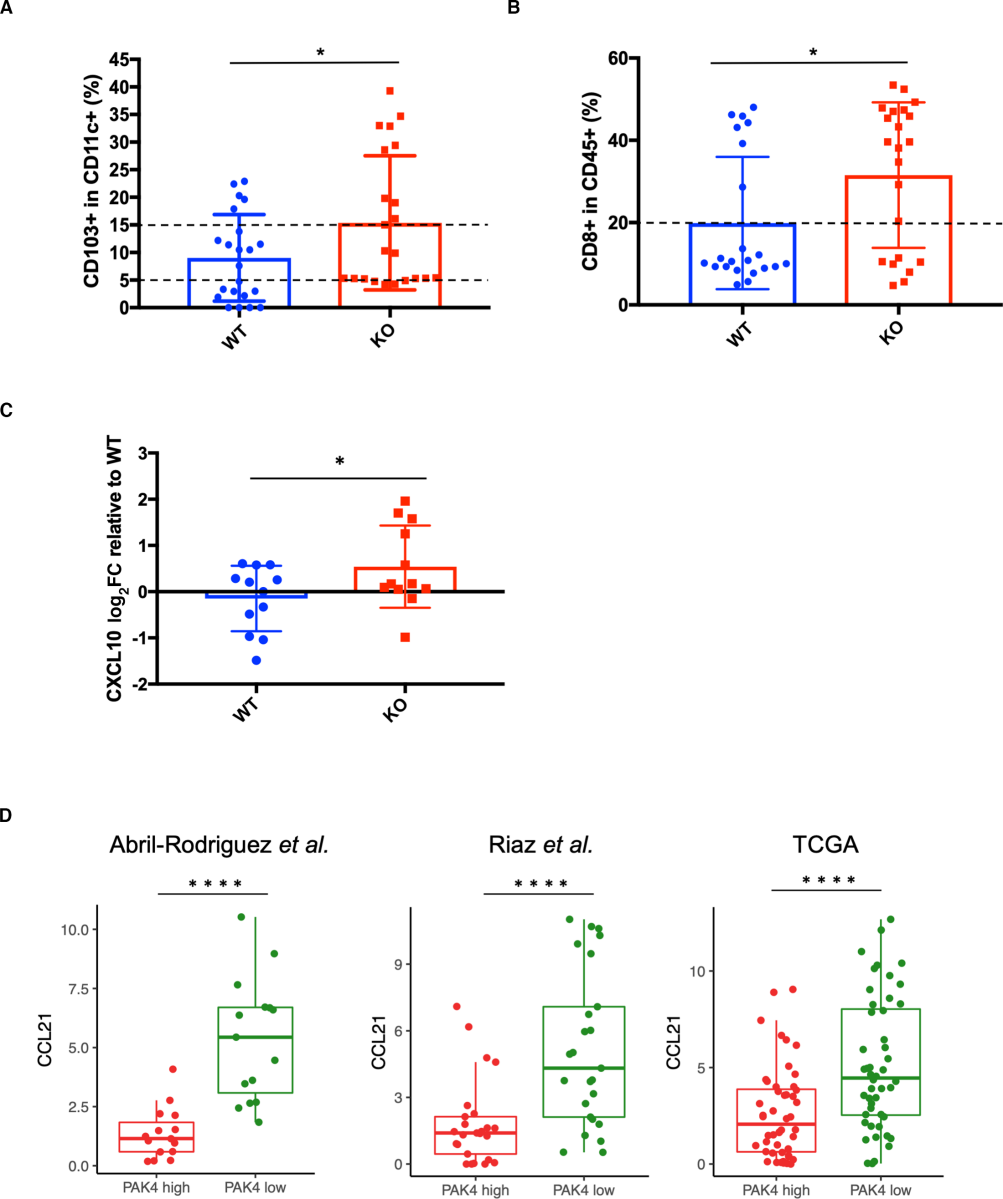
PAK4 expression levels are negatively associated with the presence of CD103^+^ DCs *in vivo* and the levels of CCR7 ligand, CCL21, in biopsies of patients with melanoma. **A,** Differences in the infiltration of CD103^+^ DCs in B16 WT CC and PAK4 KO tumors (*n* = 44, 22 per group). Tumors were collected on day 6, after one dose of anti-PD-1. After processing and staining, CD103^+^ DCs were gated for singlets, live cells, CD45^+^, MHC-II^+^, CD11c^+^, and CD103^+^ cells. B16 PAK4 KO tumors had significantly higher levels of CD103^+^ DCs compared with B16 WT CC tumors (*P* = 0.04). **B,** Differences in the infiltration of CD8^+^ cells. Samples were gated for singlets, live cells, CD45^+^, and CD8^+^ population to have an estimate of the number of CD8 cells. B16 PAK4 KO samples showed a significant increase of CD45^+^/CD8^+^ cells compared with WT CC tumors (*P* = 0.02). **C,** RNA from a total of 24 *in vivo* samples (*n* = 12 per each group) were collected to perform RT-PCR. The cycle threshold (*C*_t_) of each sample was normalized by the mean of the WT isotype group. *CXCL10* expression is significantly increased in the PAK4 KO group. **D,** Differences in *CCL21* log_2_FPKM expression levels between high and low *PAK4* in biopsies of patients with melanoma (based on the top and bottom quartile) across three different clinical datasets: Abril-Rodriguez *et al.*, Riaz *et al.*, and TCGA. In all three cohorts, *CCL21* levels were significantly enriched (*P* < 0.0001) in patients with low *PAK4* expression. Statistical significance for **D** was calculated using a two-tailed unpaired *t* test. ****, *P* < 0.0001.

To elucidate how the lack of tumor *PAK4* expression sensitizes cancer cells to anti-PD-1 immunotherapy, we aimed to characterize the tumor immune cell compartment. Previously, we described an increase in T-cell infiltration in murine melanoma B16 PAK4 KO tumors (13). In the current work, we sought to identify differences in the infiltration of other immune cell types that are key to orchestrating an antitumor response, such as DCs. To do so, we implanted B16 PAK4 KO or B16 WT CC cells in the flanks of syngeneic C57BL/6 mice and treated them with murine anti-PD-1. To study the priming of T cells in the initial steps in the generation of antitumor immunity, we harvested tumors on day 6, after administering only one dose of anti-PD-1 therapy. A total of 44 murine melanoma B16 tumors (22 PAK4 KO and 22 WT) were then analyzed by flow cytometry using specific markers to characterize DCs (Supplementary Table S1). We observed that PAK4 KO tumors, regardless of anti-PD-1 treatment, significantly increased the percentage of CD103^+^ DC (Fig. 1A), a subset of dendritic cells that have been described to excel at cross-presenting tumor antigens (31). Almost half of the WT tumors analyzed (10/22) presented less than 5% CD103^+^ DC infiltration while only three PAK4 KO tumors (3/22) had less than 5% CD103^+^ DC infiltration. In line with the increase in DC infiltration, we also observed a significant increase in the percentage of CD45^+^ CD8^+^ cells in the PAK4 KO group compared with WT tumors (Fig. 1B). Furthermore, the expression of the T-cell attracting chemokine *CXCL10*, was the only significantly enriched chemokine in PAK4 KO tumors (Fig. 1C; Supplementary Fig. S1). This is consistent with previous work in which it is shown that the expression of *CXCL10* was dependent on the presence of CD103^+^ DCs (32).

We then investigated differences in chemokine expression that could explain the increase in CD103^+^ DC levels in the tumor microenvironment. To do so, we used transcriptomic data from multiple clinical datasets (7, 13) and compared melanoma tumors with high *PAK4 expression* versus low *PAK4* expression. Interestingly, we found that among the different chemokines, high *PAK4* expression was strongly associated with decreased *CCL21* levels, which is the ligand for CCR7, the receptor expressed by CD103^+^ DC (Fig. 1D). We also found significant changes in *CCL4* expression, a chemokine that has also been reported to facilitate the infiltration of this subset of DCs (11), but to a lesser extent than *CCL21* levels (log_2_FC CCL21 = 6.09, log_2_FC CCL4 = 2.34, Abril-Rodriguez cohort). Of note, we did not find any differences in CCL21 secretion between B16 WT CC and PAK4 KO cell lines *in vitro* (Supplementary Fig. S2A) or *in vivo* (Supplementary Fig. S2B). Altogether, our results show that genetic PAK4 deletion increases the infiltration of CD103^+^ DCs, augments *CXCL10* expression and results in higher CD8 T-cell infiltration.

### Transcriptomic Characterization of PAK4 KO Cells Reveals Major Changes in the Tumor Microenvironment Followed by Increased Sensitivity to Anti-PD-1

**FIGURE 2:**
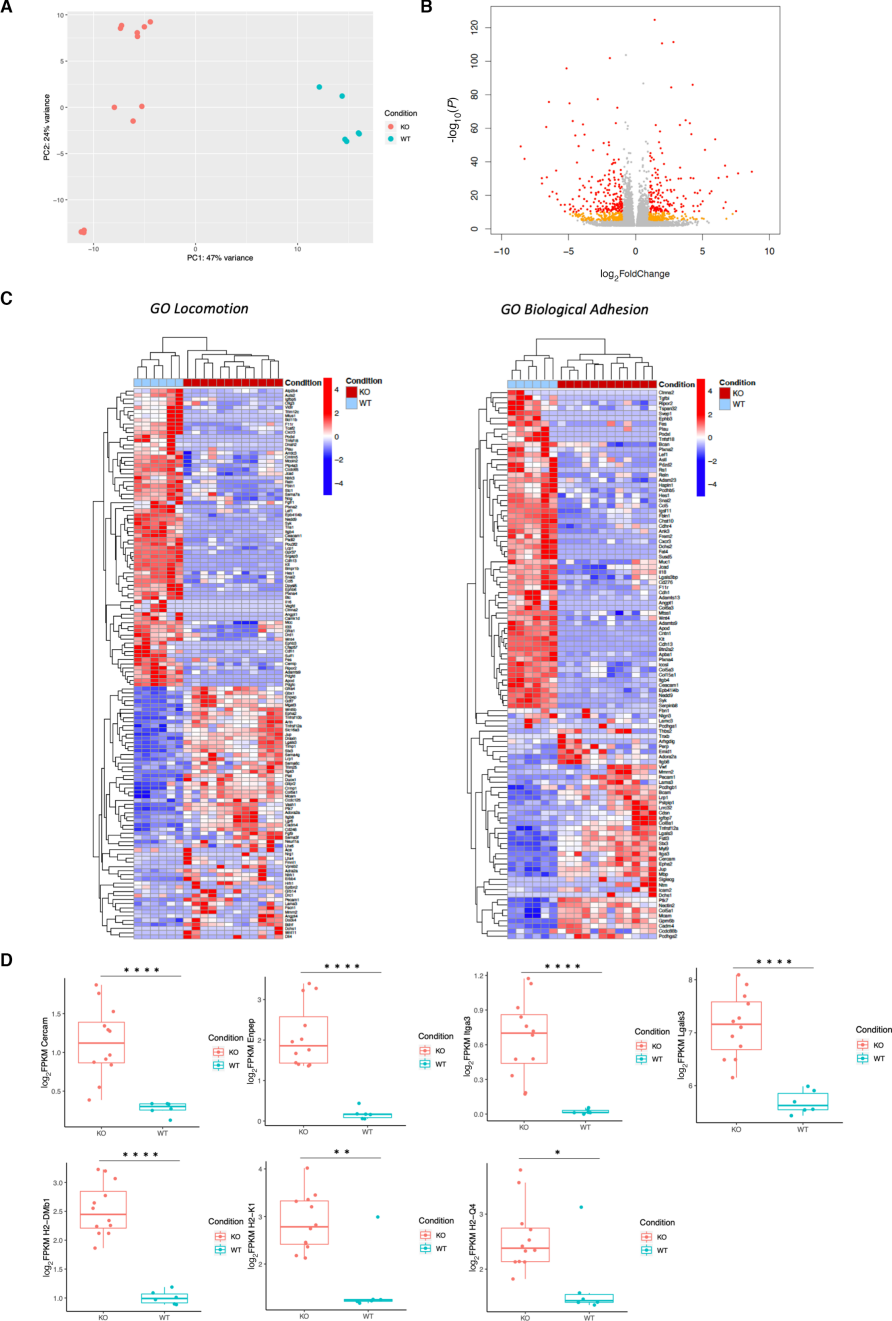
*In vitro* transcriptomic comparison of PAK4 KO and WT cells shows differences in extracellular matrix genes. **A,** PCA of 18 *in vitro* samples (12 B16 KO and 6 B16 WT CC). Principal component 1 (PC1) is related to PAK4 expression and explains almost half of the variance of this cohort (47%). **B,** Volcano plot derived from the differential gene expression analysis between B16 PAK4 KO and B16 WT CC samples. In red, genes with log_2_FC > 1 or < −1 and *P* < 5e-05. In orange, genes with log_2_FC > 1 or < −1 and *P* < 0.05. In gray, genes that do not fall in any of the two previous categories. **C,** Heatmap of two selected signatures: locomotion and biological adhesion from GO, after performing GSEA with the list of differentially expressed genes (*q* < 0.05 and log_2_FC >1 or <−1) resulted from B16 KO versus B16 WT CC comparison. Samples are separated on the basis of PAK4 expression (condition: B16 WT and B16 KO). Plotting the raw z-score. **D,** Differences between B16 PAK4 KO (*n* = 12) and B16 WT CC (*n* = 6) cells in the expression of genes associated to blood vessel formation: *Cercam* (*P* < 0.001), *Enpep* (*P* < 0.001), *Itga3* (*P* < 0.001), *Lgals3* (*P* < 0.001), and antigen presentation machinery: *H2-Dmb1* (*P* < 0.001), *H2-K1* (*P* = 0.003) and *H2-Q4* (*P* = 0.03). Statistical significance for **D** was calculated using a two-tailed unpaired *t* test. ****, *P* < 0.0001; **, *P* < 0.01; *, *P* < 0.05.

To tease out the transcriptomic differences between B16 PAK4 KO and WT cells, we performed RNA sequencing (RNA-seq) on a total of 18 *in vitro* samples (12 KO and 6 WT), which included cultures that were treated with either IFNγ, TNF, or Wnt3a. PCA showed that the main source of variance is due to lost *PAK4* expression (PC1 = 47% of variance; Fig. 2A). This was also validated by analyzing cell lines treated with IFNγ, TNF, or Wnt-3a separately, which showed that there were no significant differences on how PAK4 WT or KO cells sense through these different stimuli (Supplementary Fig. S3). Hence, these data suggest that PAK4 deletion does not sensitize tumors to IFNγ nor TNF signaling, which could contribute to improve PD-1 blockade responses. Next, to gain statistical power, we compared all KO cells versus WT regardless of any additional cytokine (Fig. 2B; Supplementary Table S2). We performed differential gene expression (DGE) analysis and used the output to perform GSEA with Gene Ontology (GO) signatures (C5), which showed that PAK4 KO cells were enriched in signatures associated with cell motility, cell adhesion, and cytoskeleton (Fig. 2C). Because of its role in cytoskeleton organization, PAK4 loss might affect how cells interact with each other, which could impact the extracellular matrix, and hence, the tumor microenvironment. Interestingly, PAK4 KO cells had a higher expression of genes associated with blood vessel formation (*Cercam1*, *Enpep*, *Itga3,* and *Lgals3*), and antigen presentation (*H2-K1* and *H2-Dmb1;*Fig. 2D). However, MHC class I surface expression analysis by flow cytometry did not show any difference between PAK4 KO and WT cells (Supplementary Fig. S4).

**FIGURE 3:**
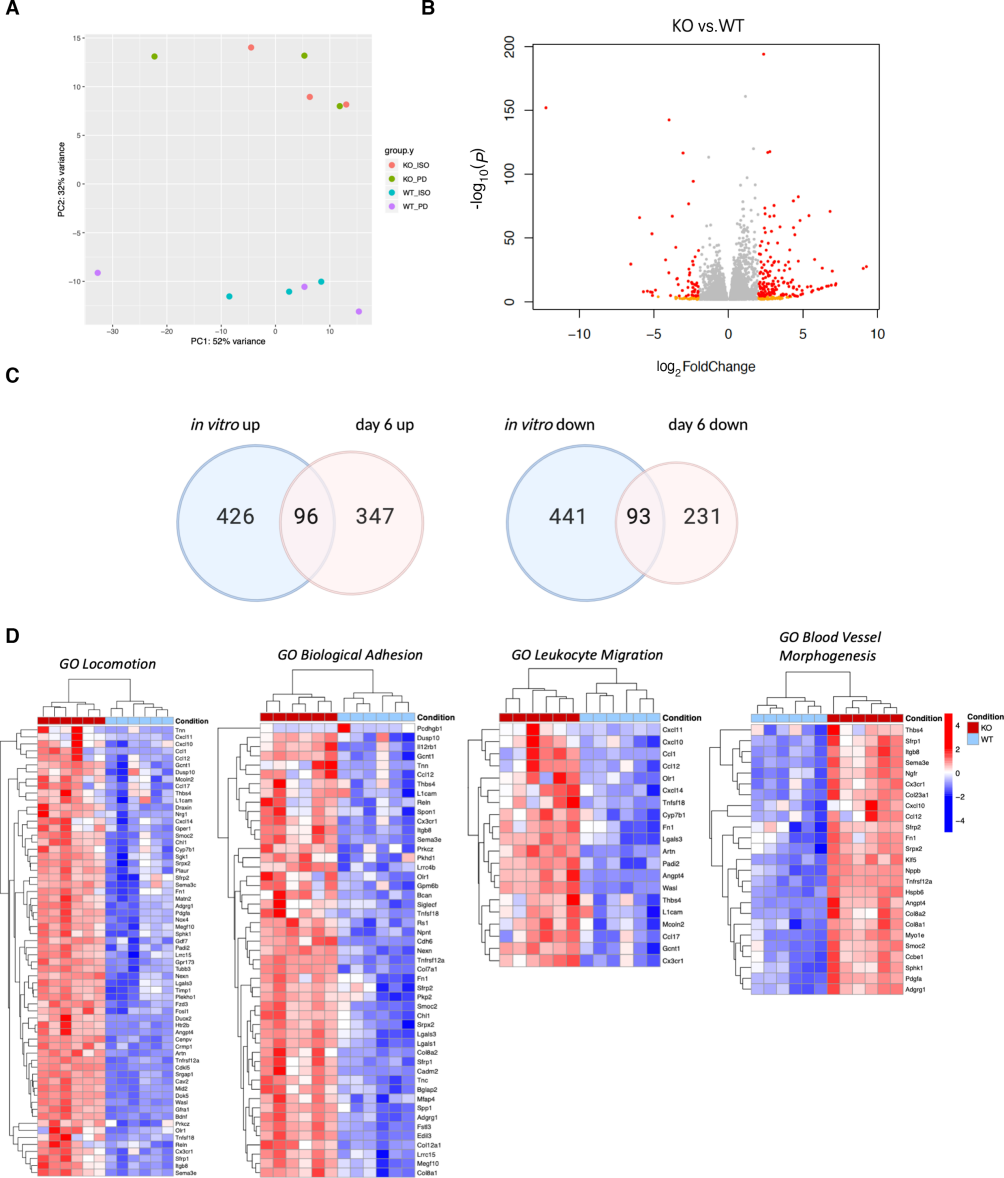
Transcriptomic characterization of early *in vivo* PAK4 KO tumors reveals major changes in the tumor microenvironment. **A,** PCA of 12 *in vivo* samples (6 B16 KO and 6 B16 WT CC). In this case, principal component 2 (PC2) is related to PAK4 expression and explains almost 32% of the variance of this cohort. **B,** Volcano plot derived from the differential gene expression analysis between B16 PAK4 KO and B16 WT CC tumors, regardless of anti-PD-1 treatment. In red, genes with log_2_FC > 1 or < −1 and *P* < 5e-05. In orange, genes with log_2_FC > 1 or < −1 and *P* < 0.05. In gray, genes that do not fall in any of the two previous categories. **C,** Venn diagram showing the overlap between DEG (*q* < 0.05 and log_2_FC >1 or <−1) *in vitro* and early *in vivo* samples. **D,** Heatmap (raw z-score) of four enriched GO signatures: locomotion, biological adhesion, leukocyte migration, and blood vessel morphogenesis, after GSEA with the DEG from comparing B16 KO versus B16 WT CC tumors. Again, samples are separated based on PAK4 expression (condition: B16 WT and B16 KO).

We next performed RNA-seq on murine melanoma B16 tumors implanted in the flanks of C57BL/6 mice that received treatment with either isotype or anti-PD-1. Here, tumors were harvested at two different timepoints: day 6 (early, one dose) and day 10 (late, three doses) so we could investigate the progression of transcriptomic changes over time. DGE analysis of day 6 tumors showed that anti-PD-1 treatment had no impact on either WT nor PAK4 KO tumors yet, as the mice had only have received one dose at this timepoint (Fig. 3A). Therefore, we focused solely on identifying the differences between WT and PAK4 KO tumors regardless of anti-PD-1 treatment status (Fig. 3B; Supplementary Table S3). We observed that 30% of the genes that were differentially expressed in PAK4 KO tumors at day 6 were also found differentially expressed in our *in vitro* analysis (Fig. 3C). B16 PAK4 KO tumors were also enriched in the same cell signatures as the PAK4 KO cells *in vitro*, such as cell motility, cell adhesion, and blood vessel morphogenesis among others (Fig. 3D). In addition, the increased expression of *Cxcl10* in PAK4 KO tumors that we have described previously, was further validated in this RNA-seq analysis (Supplementary Fig. S5).

**FIGURE 4:**
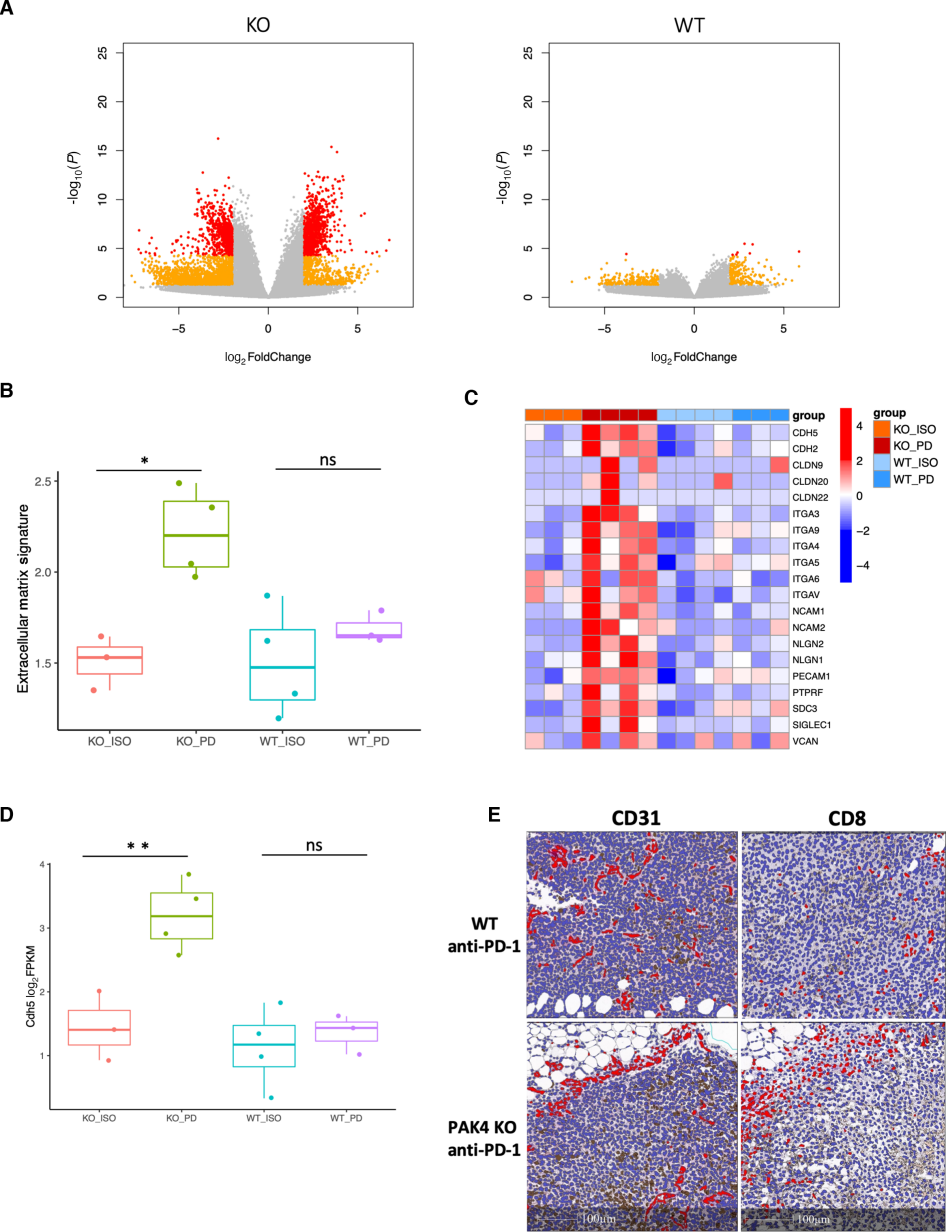
Transcriptome analysis of PAK4 KO deletion resulting in tumor sensitization to anti-PD-1 treatment. **A,** Volcano plots derived from the differential gene expression analysis between untreated and anti-PD-1 treated B16 PAK4 KO tumors (left, *n* = 7) or B16 WT CC tumors (right, *n* = 7). In red, genes with log_2_FC > 1 or < −1 and *P* < 5e-05. In orange, genes with log_2_FC > 1 or < −1 and *P* < 0.05. In gray, genes that do not fall in any of the two previous categories. **B,** Comparison of the geometric mean of a signature related to the extracellular matrix (Reactome ECM) for each of the different four groups: B16 KO Isotype (KO ISO), B16 KO anti-PD-1 (KO PD), B16 WT Isotype (WT ISO), and B16 WT anti-PD-1 (WT PD). Only B16 PAK4 KO tumors significantly change upon anti-PD-1 treatment (*P* = 0.02). **C,** Heatmap of selected genes related to the ECM such as cadherins, claudins, and integrins among others. Again, anti-PD-1 treatment affects the expression of these genes only in B16 PAK4 KO tumors (*P* = 0.008). Plotting the raw z-score. **D,** Differences between B16 PAK4 WT and B16 KO anti-PD-1 treated tumors in the expression of genes specific for endothelial cells. **E,** Images from two representative B16 WT anti-PD-1 (top) and B16 PAK4 KO anti-PD-1 (bottom) tumors. Slides were stained with CD8 and CD31. Scale bar, 100 μm. Statistical significance for **B** and **D** was calculated using one-way ANOVA and correcting for multiple comparisons using statistical hypothesis testing with the Tukey test. **, *P* < 0.01; *, *P* < 0.05.

When comparing B16 PAK4 KO and WT tumors harvested at day 10, we observed that PAK4 KO tumors underwent far more transcriptomic changes in response to anti-PD-1 (Fig. 4A). We found that only two genes changed in response to anti-PD-1 in WT tumors, which is consistent with the lack of antitumor response seen in this model, whereas up to 2,995 genes were differentially expressed in PAK4 KO tumors (log_2_FC > 2 or < −2 and FDR < 0.05; Supplementary Table S4). Therefore, lack of *PAK4* expression facilitates changes in the tumor microenvironment, which become more evident when given anti-PD-1 therapy, to sensitize melanoma B16 tumors to anti-PD-1 treatment (13). Among the differentially expressed genes, we found that the majority of changes occur in genes that are associated with or play a role in modulating the structure of the extracellular matrix (Fig. 4B). Furthermore, a cell adhesion signature with genes that play a role in the ECM and hence, could directly impact the tumor organization showed that the increase was specific for the PAK4 KO treated tumors (Fig. 4C). In addition, the comparison of PAK4 KO anti-PD-1 treated tumors with WT anti-PD-1 treated tumors yielded 2,586 genes that were differentially expressed (log_2_FC > 1 or < −1 and FDR < 0.05; Supplementary Table S5). This included several gene families that could also impact the tumor architecture, including the collagen gene family (*n* = 20), the cadherin/protocadherin gene family (*n* = 11), the integrin gene family (*n* = 8), and the adam gene family (*n* = 9) among others (Supplementary Table S6). In addition, we found that in B16 PAK4 KO tumors treated with anti-PD-1, there was an increase in the expression of a specific endothelial cell marker, *Cdh5* (Fig. 4D), indicating an increase in angiogenesis, which was already suggested in the *in vitro* and *in vivo* day 6 analyses. Of note, we performed IHC on a representative sample for each of the four groups (WT isotype, WT anti-PD-1, PAK4 KO isotype, and PAK4 KO anti-PD-1) on day 10, to evaluate the protein expression of CD8 and CD31, which is required for leukocyte transendothelial migration. We observed that PAK4 KO tumors treated with anti-PD-1 presented higher levels of both, CD31 (WT: 4% and KO: 10%) and CD8 expression (WT: 1% and KO: 9%; Fig. 4E). PAK4 KO anti-PD-1 treated tumors also showed a high level of spatial colocalization of these two markers, suggesting a proper functionality of these blood vessels and an active migration of CD8^+^ cells into the tumor (Supplementary Fig. S6). In agreement with our results, recent work by Fan and colleagues (26) demonstrated that knocking out *PAK4* in endothelial cells reorganize the whole tumor vascularity, increases immune cell infiltration and improves CAR-T cell therapy response in glioblastoma. Altogether, we characterized the tumor transcriptome of B16 PAK4 KO cells both, *in vitro* and *in vivo*, and determined that the main differences between PAK4 KO and WT transcriptomes are found in genes and cell signatures associated with the extracellular matrix.

### PAK4 Kinase Activity is Responsible for the Improved Response to Anti-PD-1 Immunotherapy *in Vivo*

**FIGURE 5:**
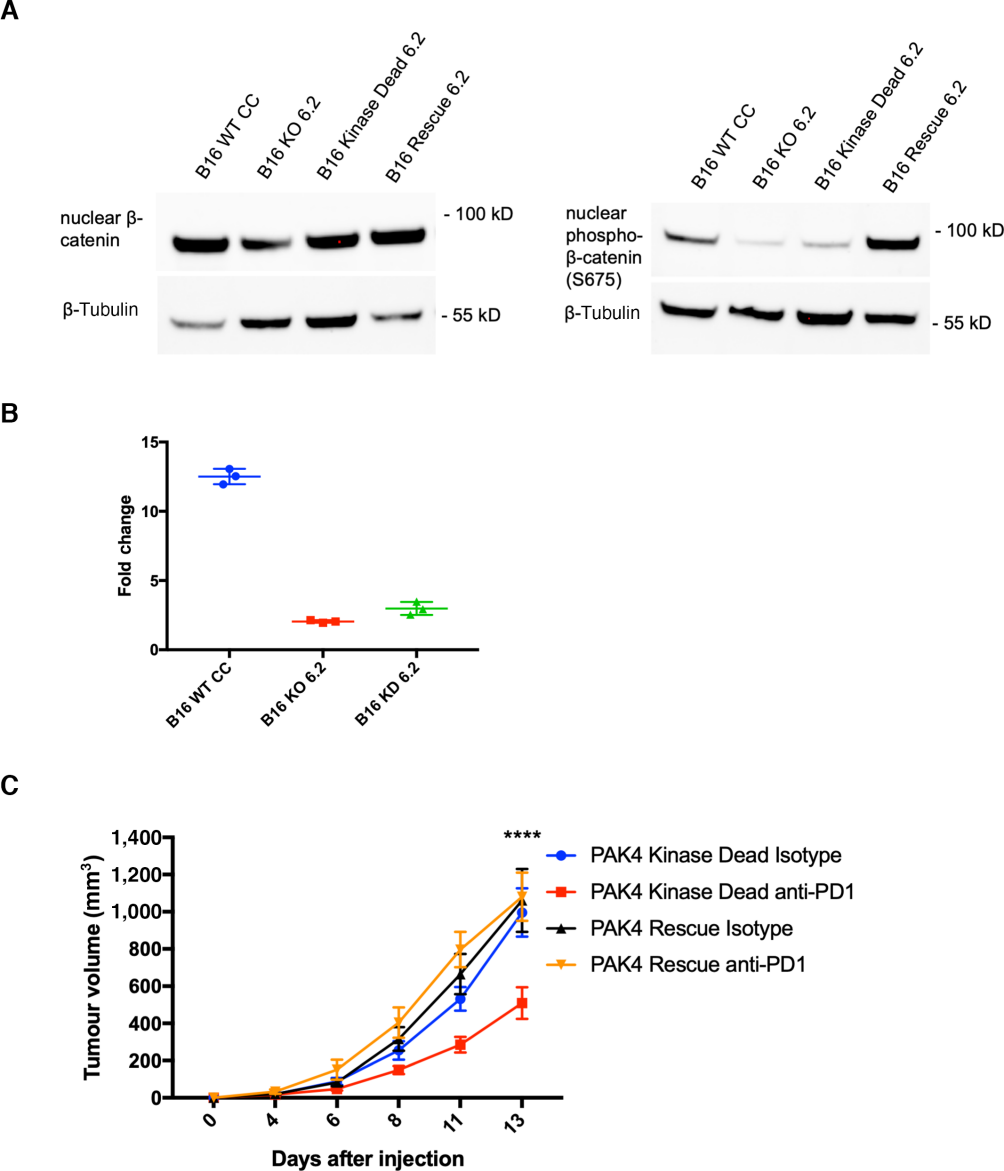
Genetic inhibition of PAK4 kinase activity sensitizes tumors to anti-PD-1 *in vivo*. **A,** Immunoblot for nuclear β-catenin expression (left) and nuclear phospho-β-catenin S675 (right) in B16 WT CC, PAK4 KO, PAK4 KD and B16 PAK4 rescue cells. PAK4 KD cells recapitulates the effects on β-catenin observed in B16 PAK4 KO cells. **B,** Topflash experiment to quantify β-catenin/WNT signaling activation. Cells were treated with ligand Wnt-3a at 200 ng/mL for 8 hours. Showing the fold change in luciferase activity between untreated and WNTA-3a treated samples for B16 WT CC, KO, and KD cells. Inhibition of PAK4 kinase activity decreases sensitivity to WNT-3a *in vitro*. Results are representative from three independent experiments. **C,** Tumor growth curves for B16 PAK4 KD tumors treated with isotype (*n* = 12, blue), anti-PD-1 (*n* = 14, red), and B16 PAK4 rescue tumors treated with isotype (*n* = 9, black) and anti-PD-1 (*n* = 9, orange). PAK4 KD tumors are able to overcome resistance to anti-PD-1 in melanoma B16 cells (*P* = 2.1e-09) day 13, comparison between PAK4 KD anti-PD-1 and rescue anti-PD-1. Statistical significance and correction for multiple comparisons was calculated using Holm-Sidak method. ****, *P* < 0.0001.

We next aimed to investigate whether inhibition of PAK4 kinase activity was sufficient to recapitulate the improved responses to ICB. Our prior work had used genetic knockdown and a PAK4 inhibitor, KPT-9274, which works by degrading the whole protein (13, 33, 34), but did not directly demonstrate that the beneficial effects of PAK4 inhibition were due to inhibition of its kinase function. To this end, we first generated three different B16 PAK4 KD cell lines by transducing PAK4 KO cells with a lentivirus containing the *PAK4* ORF with the lysine (K) at position 352 changed to a methionine (M; Supplementary Fig. S7), which was expected to inhibit PAK4 kinase activity (35, 36). To validate loss of functionality at the PAK4 kinase domain, we evaluated whether B16 PAK4 KD cells had decreased phosphorylation of β-catenin S675 and reduced response to Wnt-3a stimulation, as we have previously observed in B16 PAK4 KO cells as well as in human melanoma PAK4 KO cells (Supplementary Fig. S8). Indeed, B16 PAK4 KD cells had decreased β-catenin S675 phosphorylation (Fig. 5A) and reduced sensitivity to Wnt-3a at levels similar to the ones observed in B16 PAK4 KO cells (Fig. 5B). We next sought to determine whether B16 PAK4 KD cell lines were as sensitive to anti-PD-1 immunotherapy as B16 PAK4 KO cells. We observed that blockade of PAK4 kinase activity was sufficient to overcome resistance to anti-PD-1 therapy in B16 melanoma cells as there was a significant reduction in tumor volume in B16 PAK4 KD cells treated with anti-PD-1 compared with those treated with isotype (Fig. 5C). In summary, B16 PAK4 KD cells behave as B16 PAK4 KO cells, an observation that has important implications in the development of novel PAK4 kinase inhibitors that could potentially be more potent and specific compared with total pharmacologic PAK4 protein reduction.

### A Specific PAK4 Kinase Inhibitor Improves Responses to Anti-PD-1 Immunotherapy *in Vivo* and Resembles PAK4 KO Cells Transcriptome

**FIGURE 6:**
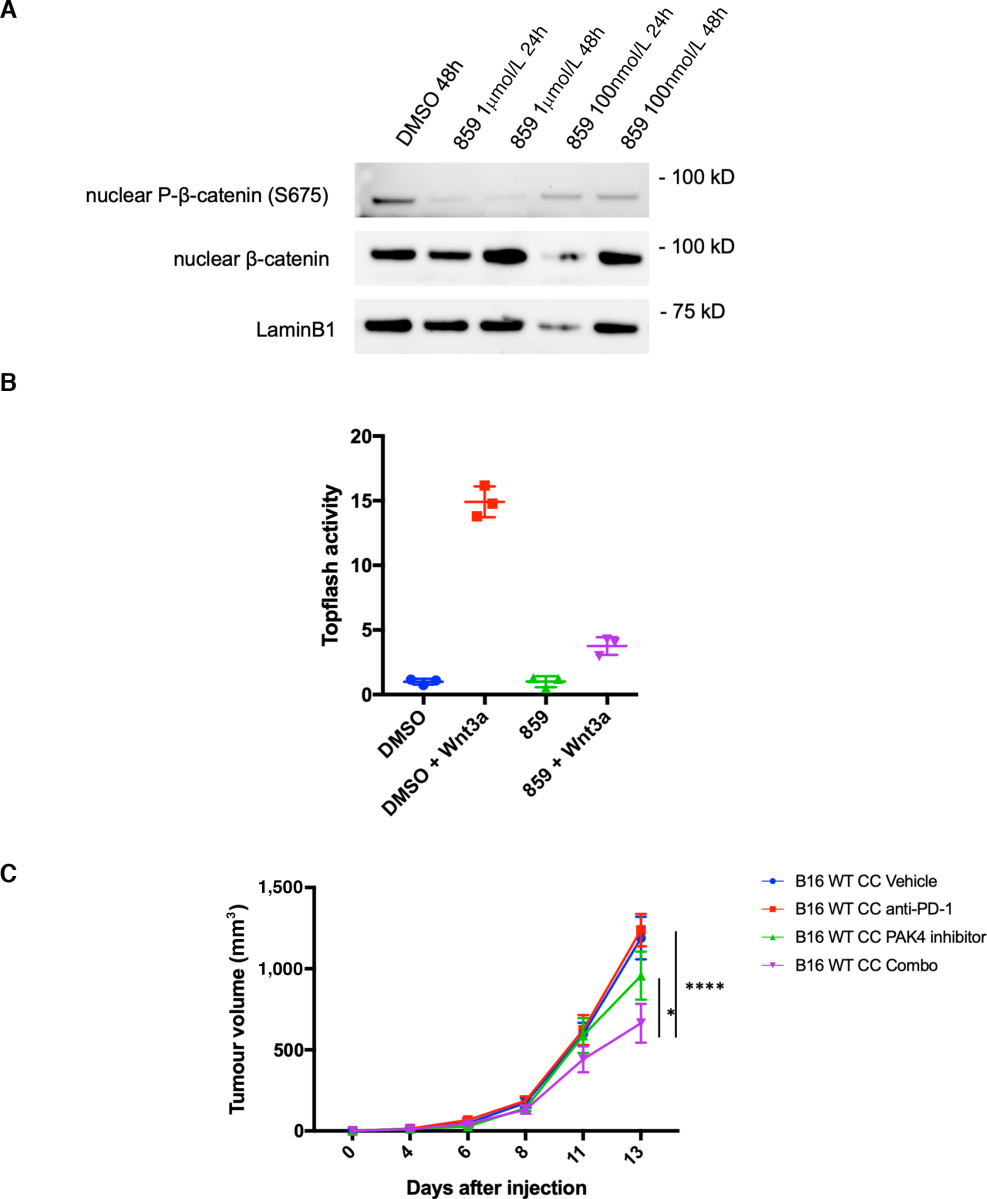
Pharmacologic inhibition of PAK4 kinase activity with A0317859 synergizes with anti-PD-1 immunotherapy *in vivo*. **A,** Immunoblot for nuclear β-catenin expression (middle lane) and nuclear phospho-β-catenin S675 (top lane) in B16 WT CC cells treated with the PAK4 inhibitor, A0317859, at two different concentrations: 1 μmol/L and 100 mmol/L, and two different timepoints 24 and 48 hours. A0317859 is able to reduce S675 β-catenin phosphorylation in all four conditions without having an impact on total nuclear β-catenin expression. **B,** Topflash experiment to test efficacy of PAK4 inhibitor on β-catenin/WNT signaling activation. The four groups include: cells treated with DMSO only, DMSO and Wnt-3a at 200 ng/mL for 8 hours, A0317859 at 1 μmol/L for 24 hours, and A0317859 at 1 μmol/L for 24 hours together with Wnt-3a at 200 ng/mL for 8 hours. A0317859 is able to reduce sensitivity to Wnt-3a stimulation as observed in B16 PAK4 KD cells. Results are representative from three independent experiments. **C,** Tumor growth curves for B16 PAK4 WT tumors treated with isotype (*n* = 8, blue), anti-PD-1 (*n* = 8, red), A0317859 (*n* = 6, green) and combination of A0317859 and anti-PD-1 (*n* = 8, purple). Pharmacologic inhibition of PAK4 kinase activity has a significantly higher antitumor activity compared with anti-PD-1 treatment alone (*P* = 3.7e-09 day 13). Statistical significance and correction for multiple comparisons was calculated using Holm-Sidak method. ****, *P* < 0.0001.

**FIGURE 7:**
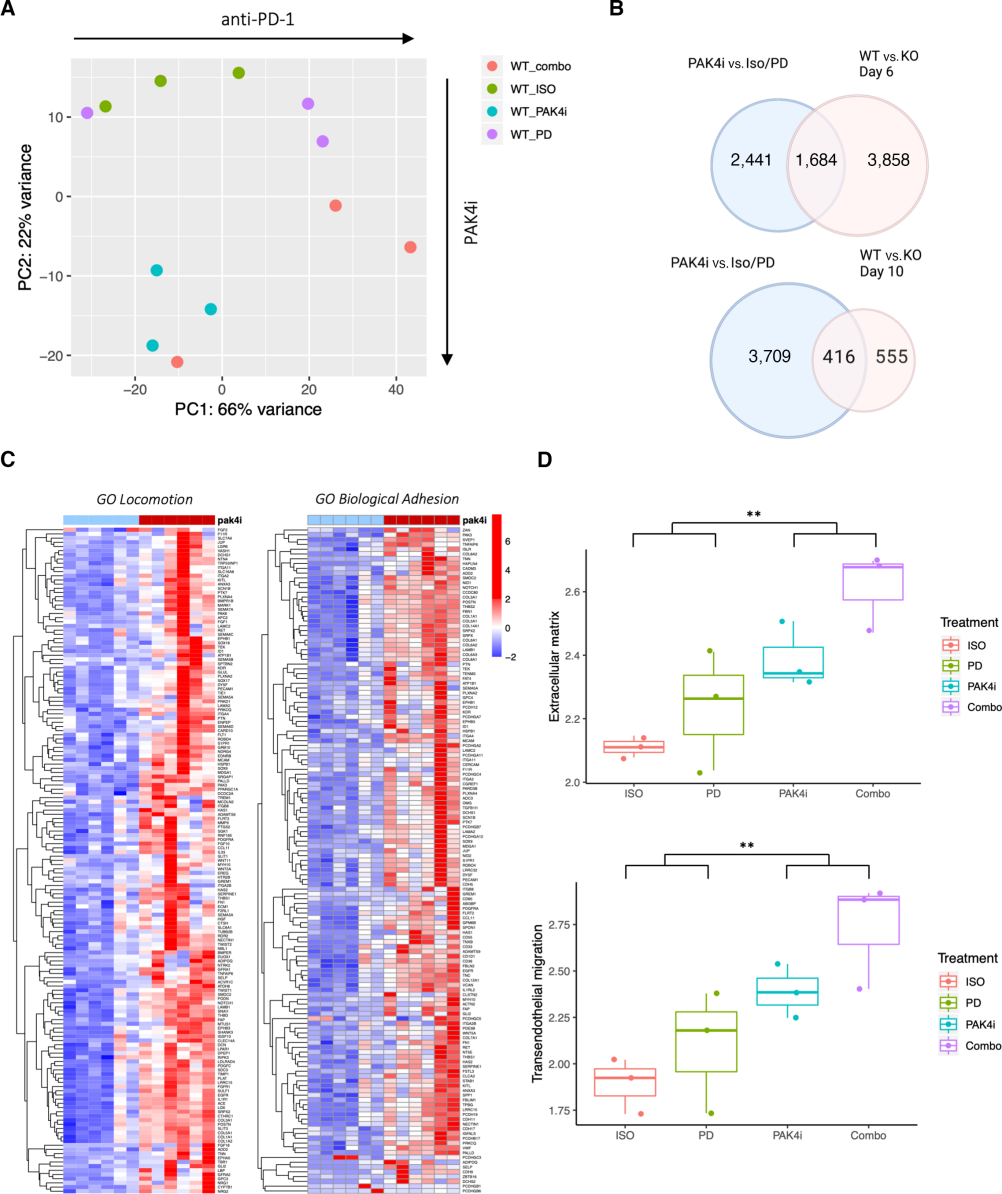
Transcriptome of tumors treated with PAK4 kinase inhibitor resemble the transcriptome of PAK4 KO cells. **A,** PCA of B16 WT tumors treated with PAK4 inhibitor (*n* = 3), isotype (*n* = 3), anti-PD-1 (*n* = 3) and combination of PAK4 inhibitor and anti-PD-1 (combo) (*n* = 3). Mice received six doses of PAK4 inhibitor and three of anti-PD-1 before harvesting tumors for RNA-seq. Principal component 1 (PC1) is related to anti-PD-1 effect and PC2 is related to the effect of PAK4 kinase inhibitor. **B,** Venn diagram showing the strong overlap between DEG (*q* < 0.05) PAK4i treated and *in vivo* samples. **C,** Heatmap (raw z-score) of two enriched GO signatures: locomotion and biological adhesion, after GSEA with the DEG from comparing PAK4i treated versus nontreated tumors. **D,** Comparison of the geometric mean of a signature related to the extracellular matrix (Reactome ECM, top, *P* = 0.0032 from comparing PAK4i treated vs. nontreated tumors) and a signature associated with transendothelial migration (bottom, *P* = 0.0068 from comparing PAK4i treated vs. nontreated tumors) for each of the different four groups: B16 WT Isotype (ISO), B16 WT anti-PD-1 (PD), B16 WT PAK4i (PAK4i), and B16 WT anti-PD-1 with PAK4i (Combo). Transendothelial migration signature consists of the following genes (*Selp*, *Sele*, *Icam1*, *Vcam1*, *Icam2*, *Cd34*, *Fn1*, *F11r, Jam2, Jam3, Pecam1*, *Cd99,* and *Cdh5*). PAK4 kinase inhibition, and especially when combined with anti-PD-1, increase the expression of both signatures. Statistical significance for **D** was calculated using a two-tailed unpaired *t* test. **, *P* < 0.01.

We next aimed to determine whether pharmacologic inhibition of PAK4 kinase activity recapitulated the effects previously observed in genetically modified cell lines. To this end, we used a novel and specific PAK4 kinase inhibitor, A0317859. First, to validate the efficacy of this compound *in vitro*, we studied its effect on the β-catenin/WNT signaling pathway. As observed in our PAK4 KO and KD cells, compound A0317859 succeeded in decreasing nuclear β-catenin phosphorylation at S675 (Fig. 6A) as well as in reducing sensitivity to Wnt-3a (Fig. 6B) in our B16 WT CC cells. We next investigated its activity *in vivo*. To do so, we treated murine melanoma B16 WT CC tumors with either vehicle, anti-PD-1, A0317859 or a combination of anti-PD-1 plus A0317859. The combination resulted in a significantly slower tumor growth compared with compound A0317859 or anti-PD-1 alone, which parallels the results of PAK4 KO and KD tumors and provides a new rationale for the combination of PD-1 blockade with a specific PAK4 kinase inhibitor (Fig. 6C). Importantly, these results were validated in an additional melanoma mouse model, YUMM2.1, in which combination of anti-PD-1 with A0317859 resulted in a significant higher antitumor activity compared to anti-PD-1 alone (Supplementary Fig. S9A and S9B). In the combination group, five of eight tumors were smaller than 500 mm^3^ at day 36, while this only occurred in one tumor of seven in the anti-PD-1 treatment. Of note, we also compared the slopes for each group using a linear mixed-effects model. Combination of both treatments resulted in a smaller slope (12.4) than anti-PD-1 alone (18.3) but it was not statistically significant (*P* = 0.073; Supplementary Fig. S9C). In addition, we characterized the transcriptome of tumors treated with the PAK4 kinase inhibitor. Interestingly, PCA showed that the two principal components of our dataset were explained by the effect of anti-PD-1 (PC1, left to right) and the effect of the PAK4i (PC2, top to bottom; Fig. 7A), which recapitulates the PCA observed in PAK4 KO versus WT cells at day 6. To further validate the activity of this novel PAK4 inhibitor, we interrogated the similarities between the differentially expressed genes (DEG) in PAK4i treated samples versus nontreated samples to the DEG in PAK4 KO versus WT samples both at days 6 and 10. We observed a 41% overlap at day 6 (Fig. 7B top), and a 43% overlap with DEG in the WT versus KO at day 10 (Fig. 7B bottom). GSEA of PAK4i-treated samples shown that tumors were also enriched in the same cell signatures as the PAK4 KO cells *in vitro* and *in vivo*, including signatures related to cell motility and cell–cell interaction (Fig. 7C). Interestingly, signatures related to WNT signaling pathway were also enriched in this group (Supplementary Fig. S10A). We found that canonical WNT/β-catenin genes were increasing, such as *Axin2* (Supplementary Fig. S10B), and others, such as *c-Myc*, were decreasing (Supplementary Fig. S10C), suggesting a complex modulation of this signaling pathway and validating the impact of blocking β-catenin phosphorylation at S675. Furthermore, the main changes in PAK4i-treated tumors occurred in genes associated with the extracellular matrix and were more pronounced when combining the kinase inhibitor with anti-PD-1 (Fig. 7D, top), as already observed in PAK4 KO tumors at day 10. Of note, we also reported changes in genes required for leukocyte transendothelial migration. We generated a signature of genes involved in this process, which included *Cdh5* and *Cd31*, and showed that tumors treated with the PAK4 kinase inhibitor, especially when combined with anti-PD-1 treatment, have an increased score (Fig. 7D, bottom). Altogether, these data strongly suggests that the PAK4 kinase inhibitor, A0317859, recapitulates the effects observed when genetically inhibiting PAK4 expression, and potentiates the immune effect of anti-PD-1 immunotherapy.

## Discussion

Several signaling pathways have been associated with resistance to ICBs, such as WNT/β-catenin signaling pathway, MAPK signaling, MYC signaling activation, pathways activated by the loss of the tumor suppressor phosphoinositide phosphatase PTEN or loss of function of liver kinase B1 (LKB1)-mediated immunosuppression (37). Ideally, these pathways could be targeted pharmacologically and used in combination with ICBs to overcome resistance to ICB therapies. Although the association of these pathways with clinical responses to immunotherapy has become more evident, there is a lack of targets that could be pharmacologically inhibited to successfully rewire these cancer-intrinsic oncogenic signaling pathways and sensitize tumors to ICB. We have previously shown that inhibition of the expression of *PAK4*, which encodes a serine-threonine kinase involved in the WNT/β-catenin pathway, increases T-cell infiltration and overcomes resistance to PD-1 blockade in several mouse models (13). However, the molecular mechanisms underlying the increased sensitivity to PD-1 blockade upon PAK4 inhibition are still unclear.

In the current study, we show that PAK4 inhibition not only increases T-cell infiltration but also increases the infiltration of a specific subset of DCs, CD103^+^ DCs. We focused on this particular subset of DCs because they have been previously associated with antitumor immunity in melanoma (11). One study showed that T-cell recruitment to the tumor was dependent on the presence of CD103^+^ DCs producing CXCL10 (32). Similarly, our results showed an increase in CD103^+^ DCs and demonstrated that *CXCL10* was the only chemokine upregulated in PAK4 KO tumors at day 6. Moreover, these changes were observed when comparing PAK4 KO versus WT tumors, regardless of anti-PD-1 treatment status, which suggests that PAK4 inhibition alters the tumor microenvironment and facilitates the infiltration of key immune cells that are required to mount an antitumor response. Nonetheless, these changes alone cannot generate a successful immune response as demonstrated by the similar growth rate of B16 PAK4 KO relative to B16 WT CC tumors. The observation that PAK4 KO tumors require the addition of anti-PD-1 to decrease tumor growth, highlights the importance of overcoming adaptive immune resistance mechanisms and blocking the PD-1/PD-L1 interaction. In line with these results, we found that *PAK4* expression negatively correlates with the expression of *CCL21*, the ligand for the CCR7 receptor expressed on CD103^+^ DC, in three independent clinical datasets. Importantly, a recent study showed that the expression of CCR7 in human melanoma correlates with the levels of T-cell infiltration and patient survival (31). The fact that PAK4 KO cells do not secrete any CCL21 suggests that, although another cell type is responsible for the secretion of this chemokine, the absence of PAK4 might contribute to increase its concentration in the tumor microenvironment. However, we could not find increased levels of CCL21 when using the B16 melanoma tumor model *in vivo*. Furthermore, we have previously described that DCs are the immune cell subtype that present the strongest negative correlation with *PAK4* expression in human melanoma tumors (13). Altogether, our results may suggest that PAK4 might play a key role in an initial step in the generation of an antitumor immune response.

The first described functional activities of PAK4 were related to cell morphology and cytoskeleton reorganization (38). Currently, PAK4 kinase activity has also been shown to regulate β-catenin phosphorylation, which impacts WNT signaling pathway activity (39). In addition, PAK4 scaffold functions include interaction with and regulation of the TNF signaling pathway. For instance, a recent study showed that PAK4 activated the TNF-survival pathway by directly facilitating the binding of TRADD to the TNF receptor (17). To tease out which functions and signaling pathways are mediating the sensitivity to anti-PD-1, in this study we performed an extensive analysis of the transcriptomic changes that occur in PAK4 KO samples in both *in vitro* and *in vivo*. From our results, we conclude that PAK4 inhibition does not alter TNF nor IFNγ signaling pathways and we also were able to exclude the possibility of increased MHC class I presentation as a mechanism of action. Our analyses validate the role of PAK4 in β-catenin phosphorylation and WNT signaling activation. However, we have yet to identify which key WNT-regulated genes are differentially stimulated in PAK4 KO cells. Therefore, the connection between PAK4, β-catenin, and ICB efficacy requires further investigation. On the other hand, our transcriptomic data support the importance of PAK4 in cell morphology, cell adhesion, and extracellular matrix organization. *In vitro*, lack of *PAK4* expression impacts the expression of genes that encode for membrane proteins involved in cell–cell interaction. Importantly, these changes are maintained in early *in vivo* samples (day 6) and with the PAK4 kinase inhibitor, and could potentially affect the tumor architecture and impact its immunogenicity, as it has been observed in other targets (40). Interestingly, we also observed changes in genes that were related to blood vessel formation. This became more relevant after Yi Fan's group demonstrated that blocking *PAK4* expression in endothelial cells could reprogram the tumor vascular microenvironment, thus, facilitating the infiltration of CAR-T cells and improving the efficacy of immunotherapy in glioblastoma (26). Although *PAK4* expression is specifically knocked out in endothelial cells, there are some similarities with our melanoma PAK4 KO cells, stressing the importance of PAK4 in modulating the tumor microenvironment and impacting immunotherapy effectiveness. For instance, our IHC analyses show an increase of CD31 in PAK4 KO anti-PD-1 treated tumors, as well as an increase of CD8, which is spatially colocalized with CD31. While this analysis does not provide any information on the quality of the blood vessels, it shows that immune cells are able to infiltrate the tumor through them, suggesting that the blood vessels are functional. Of note, these results are limited by the sample size. Furthermore, the importance of PAK4 deletion is also supported by the PAK4 inhibitor as well as the late *in vivo* RNA-seq data, where anti-PD-1 treatment only changed the transcriptome of melanoma PAK4 KO tumors. In both cases, the main differences were observed in genes related to the extracellular matrix and angiogenesis, and are accompanied by an improved response to ICB in our B16 melanoma mouse model. Nevertheless, whilst the association between PAK4 and the tumor microenvironment is evident, further studies are needed to elucidate the key changes that facilitate the infiltration of immune cells and overcome the resistance to ICB.

The identification of oncogenic-driven resistance mechanisms to ICB can provide novel candidates for clinical intervention. However, discovering targets that could be exploited pharmacologically remains a challenge. Specifically, the β-catenin/WNT signaling pathway has been extensively associated with poor immune infiltration and lack of response to PD-1 blockade, but to date, there are no clinical trials that have successfully combined WNT-inhibitors with ICB inhibitors. This is in part due to the complexity and importance of this signaling pathway in regulating several essential cellular functions, which could narrow the therapeutic window. Interestingly, the novel PAK4 kinase inhibitor tested here has a clear impact on WNT/β-catenin signaling pathway, with the expression of genes involved in WNT canonical signaling being significantly affected. Our results open the possibility that the modulation of WNT signaling through the blockade of S675 β-catenin phosphorylation could sensitize tumors to anti-PD-1 immunotherapy. However, although the link between PAK4 and β-catenin is consistent in our model, we need to further investigate how PAK4 inhibition rewires the WNT signaling pathway and examine how these changes impact anti-PD1 efficacy. Our results demonstrate that lack of *PAK4* expression modifies the sensitivity to the main WNT ligand, Wnt-3a, while other WNT-dependent cellular functions, such as cell proliferation, remain intact. Importantly, we demonstrate that PAK4 kinase function is responsible for both: WNT signaling alteration and increased sensitivity to PD-1 blockade immunotherapy. Yet, we cannot completely exclude the possibility that other kinase-independent PAK4 functions may contribute to the phenotype we observe *in vivo*. Previous attempts to block PAK4 activity pharmacologically have failed due to a dearth of selective inhibitors or due to issues with the compound pharmacokinetics, as observed in the terminated clinical trial evaluating the pan-PAK inhibitor, PF-03758309 (41). Here, we provide the initial results of a specific and novel PAK4 kinase inhibitor, A0317859. Importantly, this compound is able to recapitulate the *in vivo* efficacy observed in our B16 PAK4 KO and PAK4 KD *in vivo* models as well as resemble the transcriptome of PAK4 KO cells.

In summary, in this study we showed how PAK4 inhibition remodels the tumor microenvironment, enabling the infiltration of key immune cell subtypes and changing the expression of genes involved in the tumor architecture. In addition, we established that blocking PAK4 kinase function is sufficient to overcome PD-1 blockade resistance *in vivo* and demonstrated how a novel PAK4 kinase inhibitor could potentially overcome resistance to PD-1 blockade*.* To date, there is only a single clinical trial (NCT02702492), combining an anti-PD-1 antibody with a dual PAK4 and NAMPT inhibitor, KPT-9274, which decreases whole PAK4 protein expression. Our work lays the foundation for the translation of a novel, unique and specific PAK4 kinase inhibitor that could be used in combination with PD-1 blockade immunotherapy.

## Supplementary Material

Supplementary Figure 1 Supplementary Figure 1: Impact of PAK4 deletion on the expression of chemokines.

Supplementary Figure 2 Supplementary Figure 2: PAK4 deletion does not affect CCL21 secretion.

Supplementary Figure 3 Supplementary Figure 3: No difference in signalling through different stimuli between PAK4 KO and WT cells.

Supplementary Figure 4 Supplementary Figure 4: PAK4 deletion does not affect MHC-I and II surface expression.

Supplementary Figure 5 Supplementary Figure 5: PAK4 deletion increases CXCL10 expression in vivo.

Supplementary Figure 6 Supplementary Figure 6: Spatial colocalization of CD8 and CD31 positive cells.

Supplementary Figure 7 Supplementary Figure 7: PAK4 protein expression levels in the different cell lines.

Supplementary Figure 8 Supplementary Figure 8: PAK4 KO in human melanoma cells impairs B-catenin/WNT signalling.

Supplementary Figure 9 Supplementary Figure 9: PAK4 inhibitor, A0317859, also synergizes with PD-1 blockade in YUMM2.1 melanoma cells.

Supplementary Figure 10 Supplementary Figure 10: PAK4 kinase inhibition modulates WNT signalling in vivo.

Supplementary Table 1 Supplementary Table 1: Panel of immune markers for dendritic cell characterization.

Supplementary Table 2 Supplemental Table 2: Differential gene expression analysis between B16 KO and WT cells in vitro. Showing genes with q < 0.05 and log2FC > 1 or < -1.

Supplementary Table 3 Supplemental Table 3: Differential gene expression analysis between B16 KO and WT tumors in vivo at day 6. Showing genes with q < 0.05 and log2FC > 1 or < -1.

Supplementary Table 4 Supplemental Table 4: Differential gene expression analysis between B16 PAK4 KO anti-PD-1 vs PAK4 KO isotype tumors in vivo at day 10. Showing genes with q < 0.05 and log2FC > 2 or < -2.

Supplementary Table 5 Supplemental Table 5: Differential gene expression analysis between B16 PAK4 KO anti-PD-1 vs WT anti-PD-1 tumors in vivo at day 10. Showing genes with q < 0.05 and log2FC > 1 or < -1.

Supplementary Table 6 Supplemental Table 6: List of extracellular matrix genes enriched in PAK4 KO anti-PD-1 treated samples.
