# Supplementary material for: Remodeling of the Tumor Microenvironment Through PAK4 Inhibition Sensitizes Tumors to Immune Checkpoint Blockade
Source: Cancer Res Commun. 2022 Oct 19;2(10):1214–28. doi: 10.1158/2767-9764.CRC-21-0133 (PMC9799984; doi:10.1158/2767-9764.CRC-21-0133)
Supplement: Supplementary Figure 1 — Impact of PAK4 deletion on the expression of chemokines. [file crc-21-0133-s01.pdf]

Supplementary Fig. S1

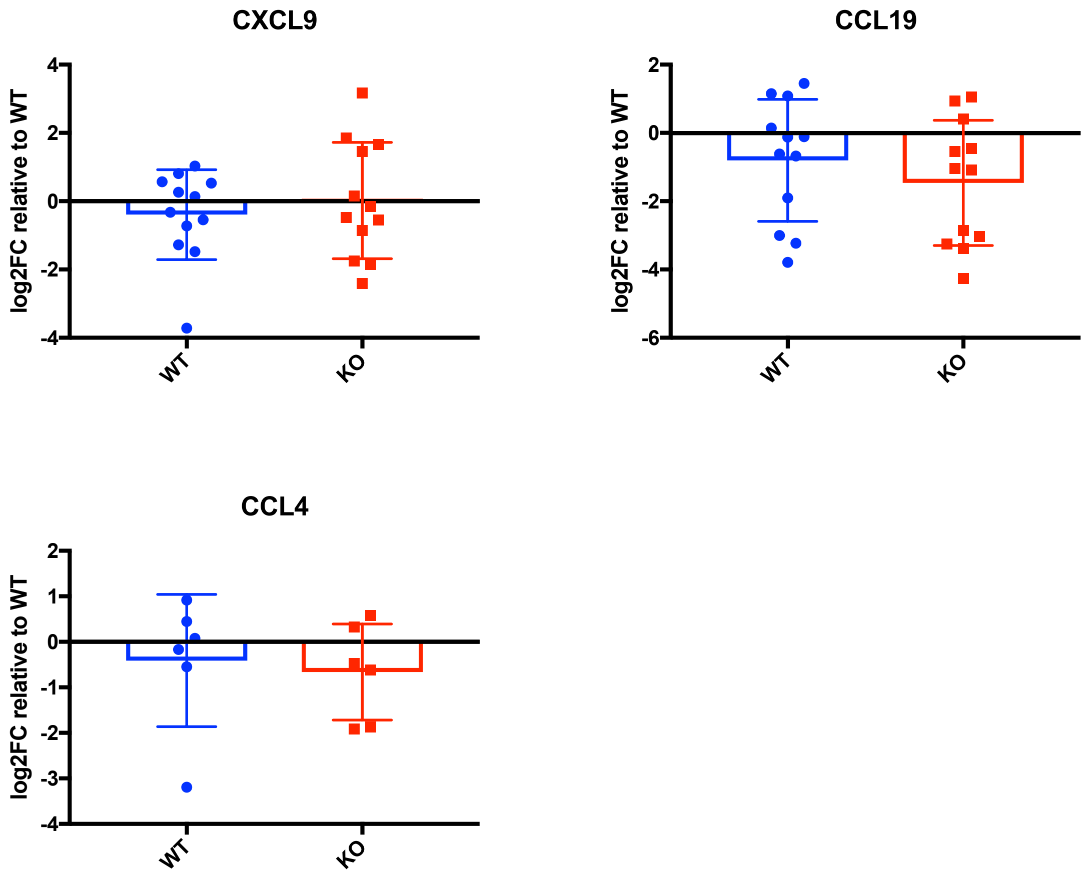

Supplementary Figure 1: Impact of PAK4 deletion on the expression of chemokines . RNA from a total of 24 in vivo samples (n = 12 per each group) were collected to perform RT-PCR. The cycle threshold (Ct) of each sample was normalized by the mean of the WT isotype group. The expression of CXCL9, CCL19 and CCL4 is not significantly increased in the PAK4 KO group.
