# Supplementary material for: Remodeling of the Tumor Microenvironment Through PAK4 Inhibition Sensitizes Tumors to Immune Checkpoint Blockade
Source: Cancer Res Commun. 2022 Oct 19;2(10):1214–28. doi: 10.1158/2767-9764.CRC-21-0133 (PMC9799984; doi:10.1158/2767-9764.CRC-21-0133)
Supplement: Supplementary Figure 2 — PAK4 deletion does not affect CCL21 secretion. [file crc-21-0133-s02.pdf]

Supplementary Fig. S2

a

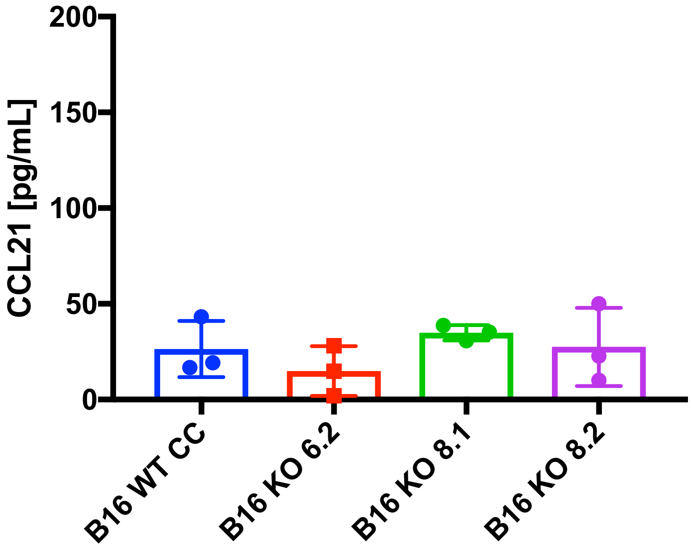

b

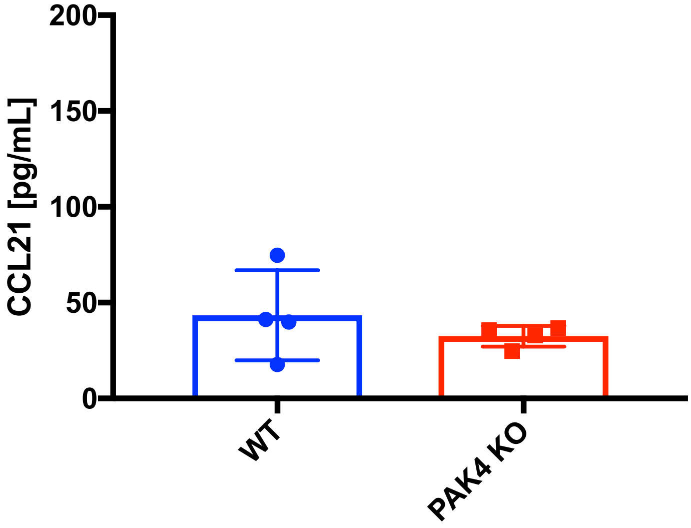

Supplementary Figure 2: PAK4 deletion does not affect CCL21 secretion. a, Cells were seeded in 96 well plates by triplicate and media from B16 WT CC and three independent PAK4 KO cell lines were collected to measure CCL21 protein levels by ELISA. **b,** *In vivo* B16 WT and PAK4 KO tumors were harvested and homogenised to measure CCL21 protein levels by ELISA. No differences between WT and KO cells were observed *in vitro* or *in vivo*.
