# Supplementary material for: Remodeling of the Tumor Microenvironment Through PAK4 Inhibition Sensitizes Tumors to Immune Checkpoint Blockade
Source: Cancer Res Commun. 2022 Oct 19;2(10):1214–28. doi: 10.1158/2767-9764.CRC-21-0133 (PMC9799984; doi:10.1158/2767-9764.CRC-21-0133)
Supplement: Supplementary Figure 3 — No difference in signalling through different stimuli between PAK4 KO and WT cells. [file crc-21-0133-s03.pdf]

Supplementary Fig. S3

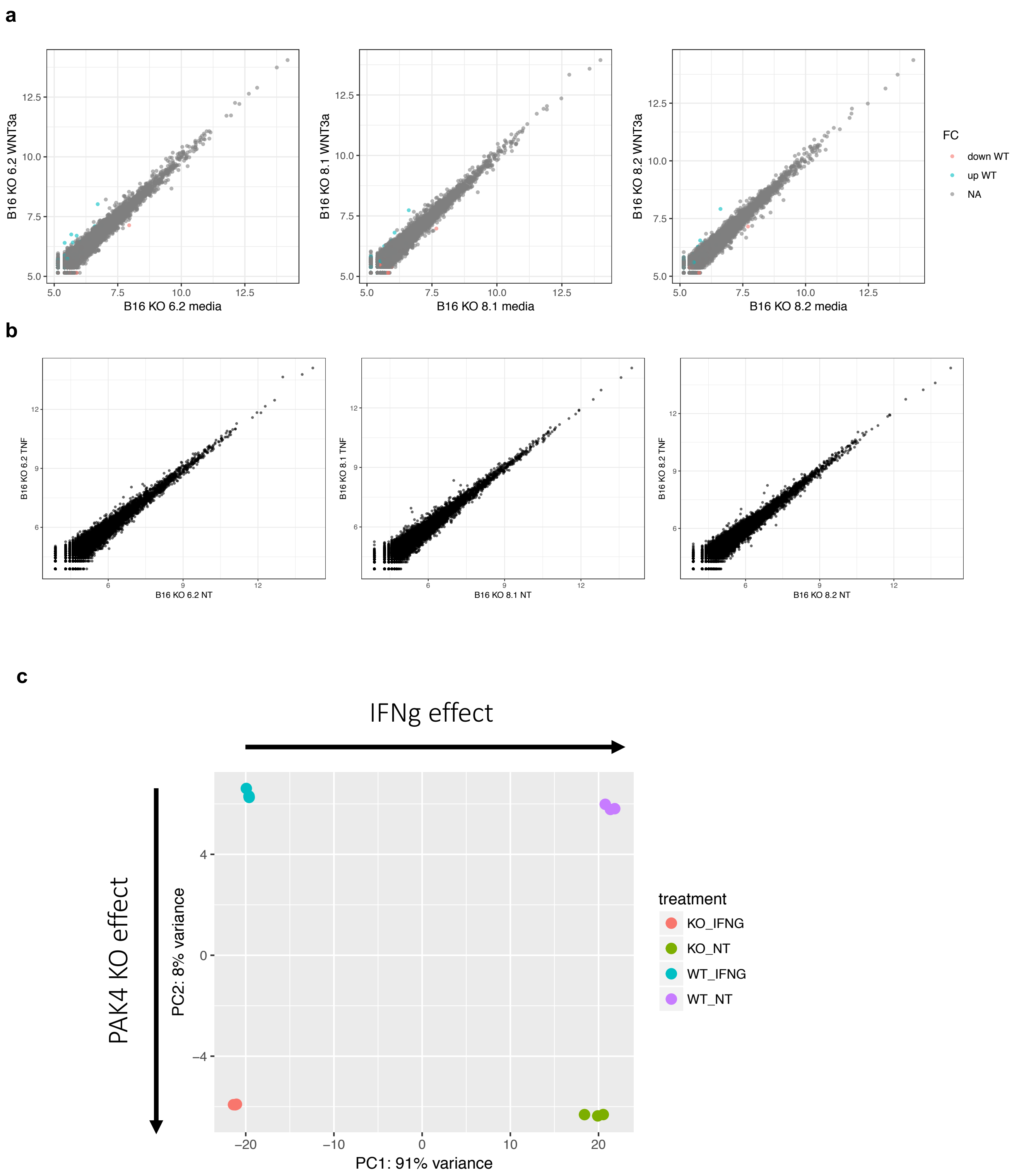

Supplementary Figure 3: No difference in signalling through different stimuli between PAK4 KO and WT cells. a, We collected RNA and sequenced cells treated with Wnt-3a at 200ng/mL for 8 hours. Scatter plot of the log2FPKM expression of all genes between untreated (X axis) and treated (Y axis) cells for each of the different PAK4 KO clones. To visualize that the same genes that change upon Wnt-3a stimuli in KO cells, also change in WT cells, we coloured them in red (up-regulated) and blue (down-regulated). **b,** Cells were treated with TNF at 100ng/mL for 6 hours before extracting RNA for sequencing. As in a, showing scatter plot of the log2FPKM expression of all genes between untreated and treated cells. TNF does not affect the transcriptome of B16 PAK4 KO cells. **c,** We collected RNA and sequenced cells treated with IFNg at 100ng/mL for 6 hours. Principal component analysis reveals that there are no differences in IFNg signalling between PAK4 KO and WT cells as both fall in the same position of the PC1 axis which explains the effect of adding IFNg.
