# Supplementary material for: Remodeling of the Tumor Microenvironment Through PAK4 Inhibition Sensitizes Tumors to Immune Checkpoint Blockade
Source: Cancer Res Commun. 2022 Oct 19;2(10):1214–28. doi: 10.1158/2767-9764.CRC-21-0133 (PMC9799984; doi:10.1158/2767-9764.CRC-21-0133)
Supplement: Supplementary Figure 4 — PAK4 deletion does not affect MHC-I and II surface expression. [file crc-21-0133-s04.pdf]

Supplementary Fig. S4

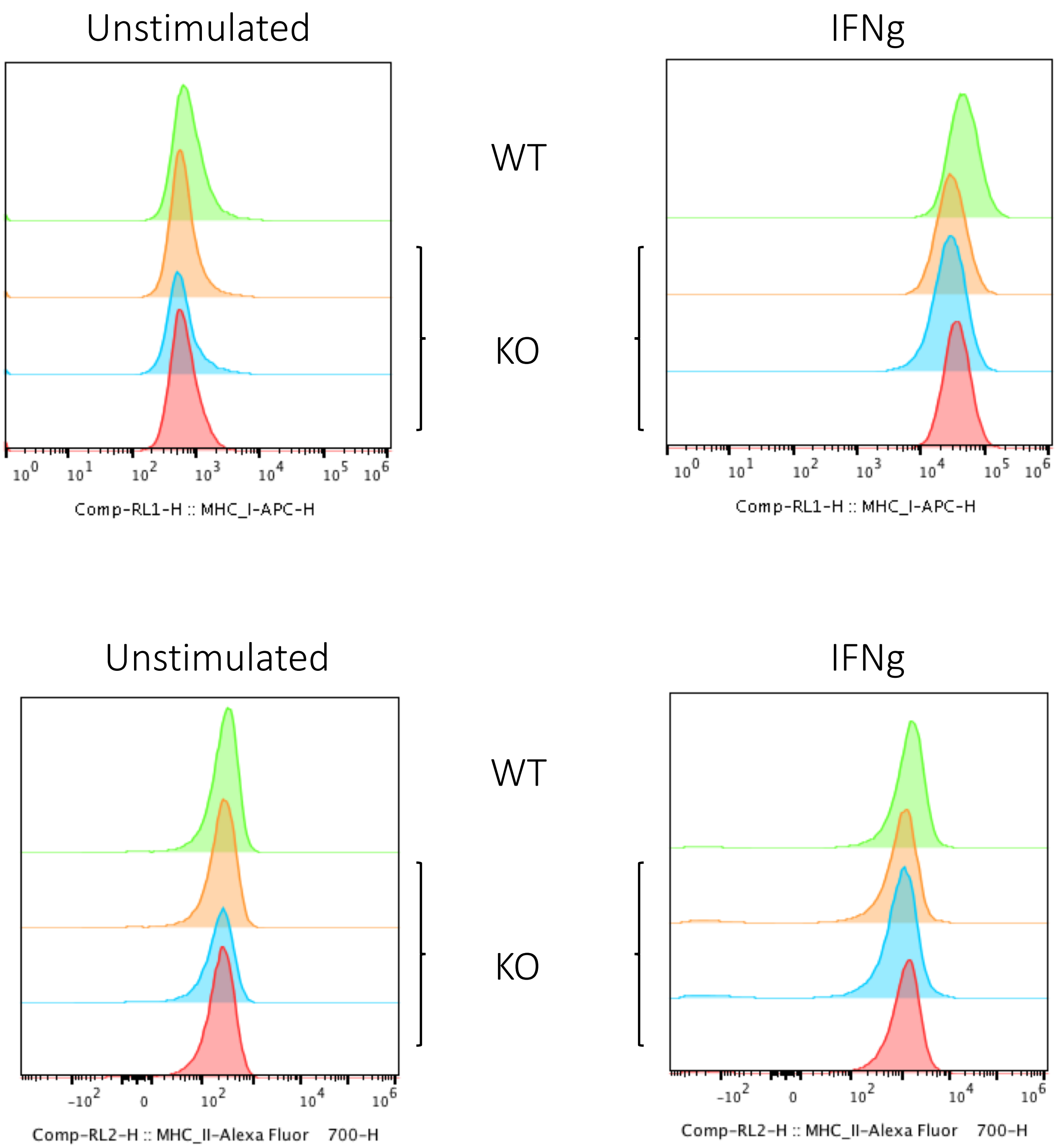

Supplementary Figure 4: PAK4 deletion does not affect MHC-I and II surface expression. B16 WT and KO cells were treated with IFNg at 100ng/mL for 6 hours and stained for MHC-I and II expression. No statistical differences between the MHC surface up-regulation in WT and KO cells was found.
