# Supplementary material for: Remodeling of the Tumor Microenvironment Through PAK4 Inhibition Sensitizes Tumors to Immune Checkpoint Blockade
Source: Cancer Res Commun. 2022 Oct 19;2(10):1214–28. doi: 10.1158/2767-9764.CRC-21-0133 (PMC9799984; doi:10.1158/2767-9764.CRC-21-0133)
Supplement: Supplementary Figure 5 — PAK4 deletion increases CXCL10 expression in vivo. [file crc-21-0133-s05.pdf]

Supplementary Fig. S5

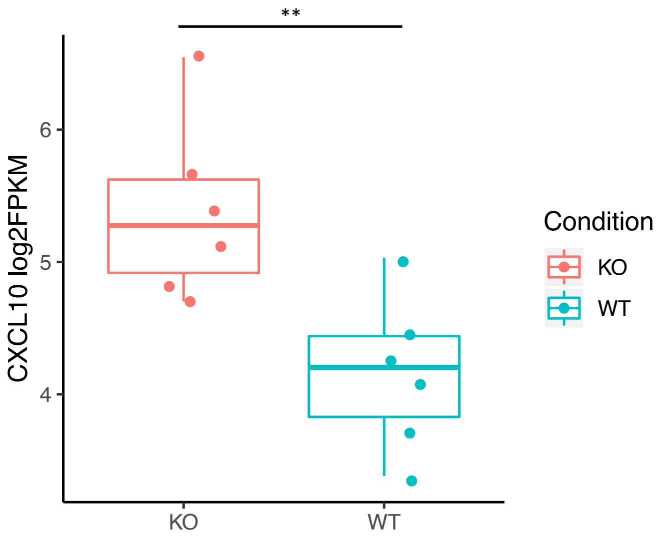

Supplementary Figure 5: PAK4 deletion increases CXCL10 expression *in vivo*. Boxplot showing the log2FPKM for CXCL10 at day 6 in PAK4 KO tumors (n= 6) and WT tumors (n = 6). CXCL10 expression in enriched in KO tumors. Statistical significance was calculated using a two-tailed un-paired t-test. **P < 0.01
