# Supplementary material for: Remodeling of the Tumor Microenvironment Through PAK4 Inhibition Sensitizes Tumors to Immune Checkpoint Blockade
Source: Cancer Res Commun. 2022 Oct 19;2(10):1214–28. doi: 10.1158/2767-9764.CRC-21-0133 (PMC9799984; doi:10.1158/2767-9764.CRC-21-0133)
Supplement: Supplementary Figure 6 — Spatial colocalization of CD8 and CD31 positive cells. [file crc-21-0133-s06.pdf]

Supplementary Fig. S6

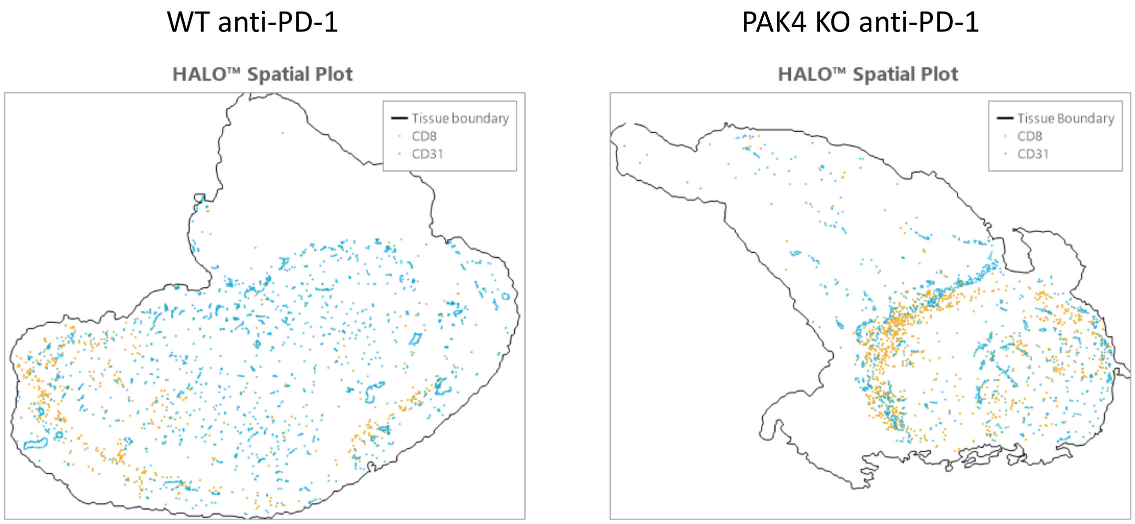

Supplementary Figure 6: Spatial colocalization of CD8 and CD31 positive cells. PAK4 KO tumors treated with anti-PD-1 show a high overlap between CD8 and CD31 while WT anti-PD-1 tumors show a more diffuse CD31 distribution without $CD8^+/CD31^+$ clusters.
