# Supplementary material for: Remodeling of the Tumor Microenvironment Through PAK4 Inhibition Sensitizes Tumors to Immune Checkpoint Blockade
Source: Cancer Res Commun. 2022 Oct 19;2(10):1214–28. doi: 10.1158/2767-9764.CRC-21-0133 (PMC9799984; doi:10.1158/2767-9764.CRC-21-0133)
Supplement: Supplementary Figure 7 — PAK4 protein expression levels in the different cell lines. [file crc-21-0133-s07.pdf]

Supplementary Fig. S7

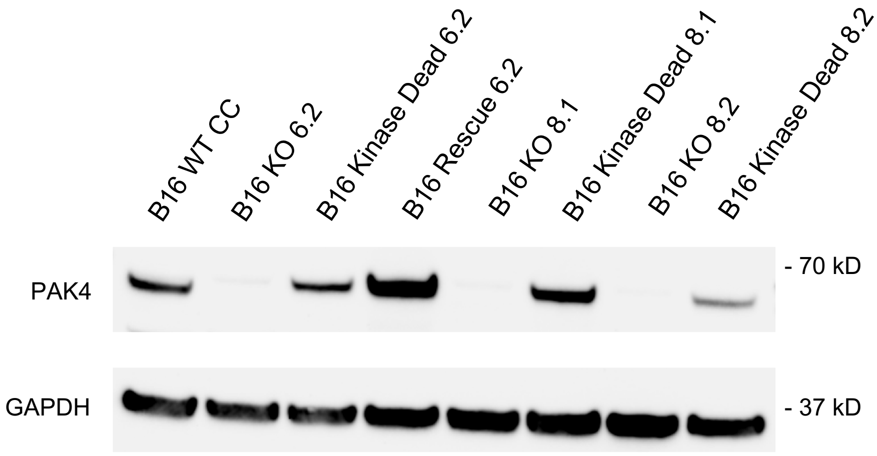

Supplementary Figure 7: PAK4 protein expression levels in the different cell lines. Cells were cultured and harvested for protein isolation upon reaching 80% confluency. Showing immunoblot for PAK4 expression protein levels in WT, PAK4 KO, Kinase Dead and Rescue cell lines.
