# Supplementary material for: Remodeling of the Tumor Microenvironment Through PAK4 Inhibition Sensitizes Tumors to Immune Checkpoint Blockade
Source: Cancer Res Commun. 2022 Oct 19;2(10):1214–28. doi: 10.1158/2767-9764.CRC-21-0133 (PMC9799984; doi:10.1158/2767-9764.CRC-21-0133)
Supplement: Supplementary Figure 8 — PAK4 KO in human melanoma cells impairs B-catenin/WNT signalling. [file crc-21-0133-s08.pdf]

Supplementary Fig. S8

a

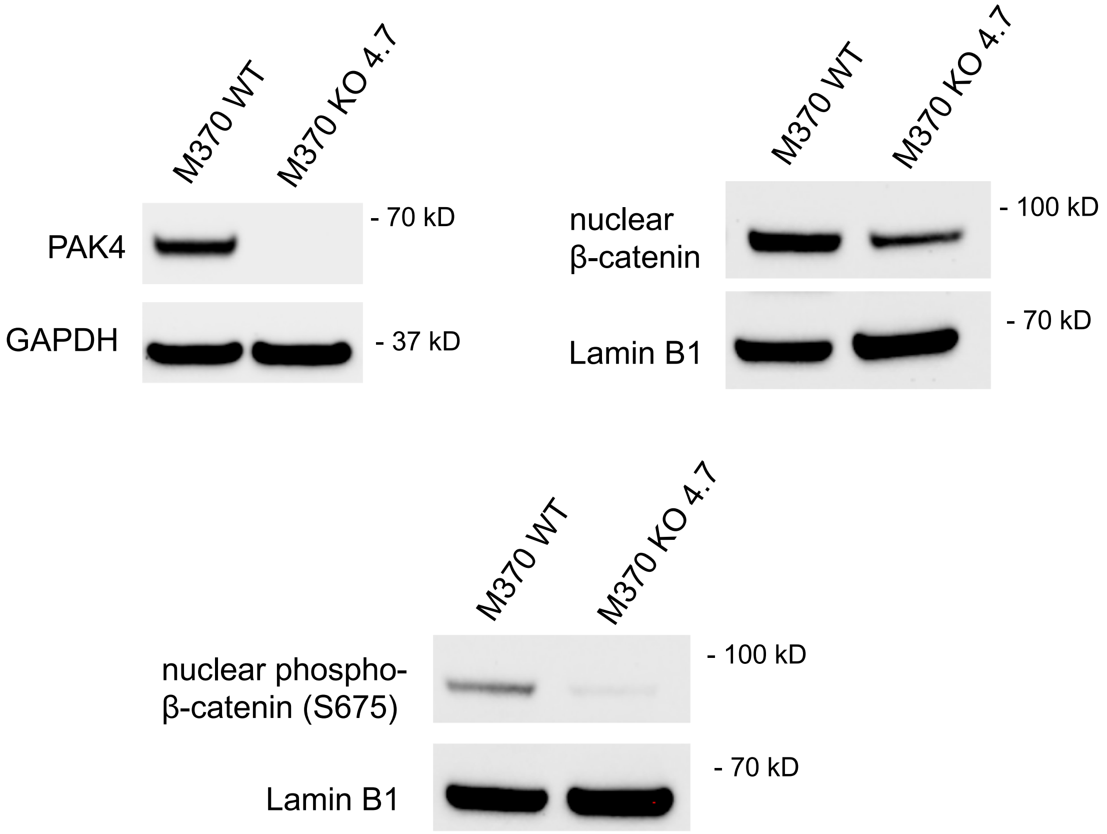

b

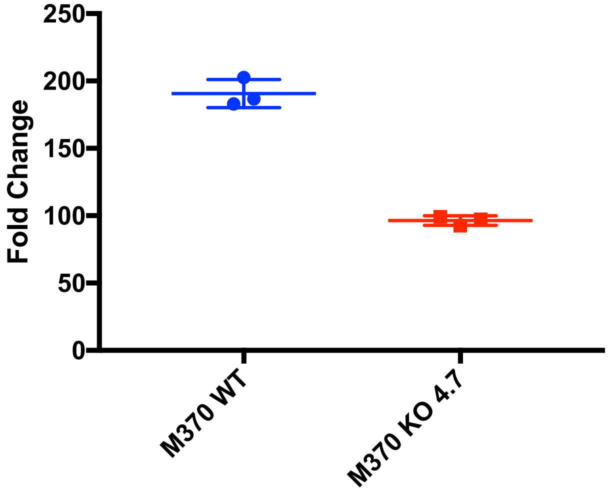

Supplementary Figure 8: PAK4 KO in human melanoma cells impairs B-catenin/WNT signalling. a, Immunoblot for PAK4, nuclear B-catenin and S675 phospho-B-catenin in human melanoma M370 cells. **b,** M370 WT and KO cells were treated with Wnt-3a at 200ng/mL for 8 hours to perform a Topflash assay. Showing the fold change between Wnt-3a treated and untreated cells for both groups. As observed in B16 PAK4 KO cells, PAK4 deletion decreases S675 phosphorylation and reduces sensitivity to Wnt-3a stimulation.
