# Supplementary material for: Remodeling of the Tumor Microenvironment Through PAK4 Inhibition Sensitizes Tumors to Immune Checkpoint Blockade
Source: Cancer Res Commun. 2022 Oct 19;2(10):1214–28. doi: 10.1158/2767-9764.CRC-21-0133 (PMC9799984; doi:10.1158/2767-9764.CRC-21-0133)
Supplement: Supplementary Figure 9 — PAK4 inhibitor, A0317859, also synergizes with PD-1 blockade in YUMM2.1 melanoma cells. [file crc-21-0133-s09.pdf]

Supplementary Fig. S9

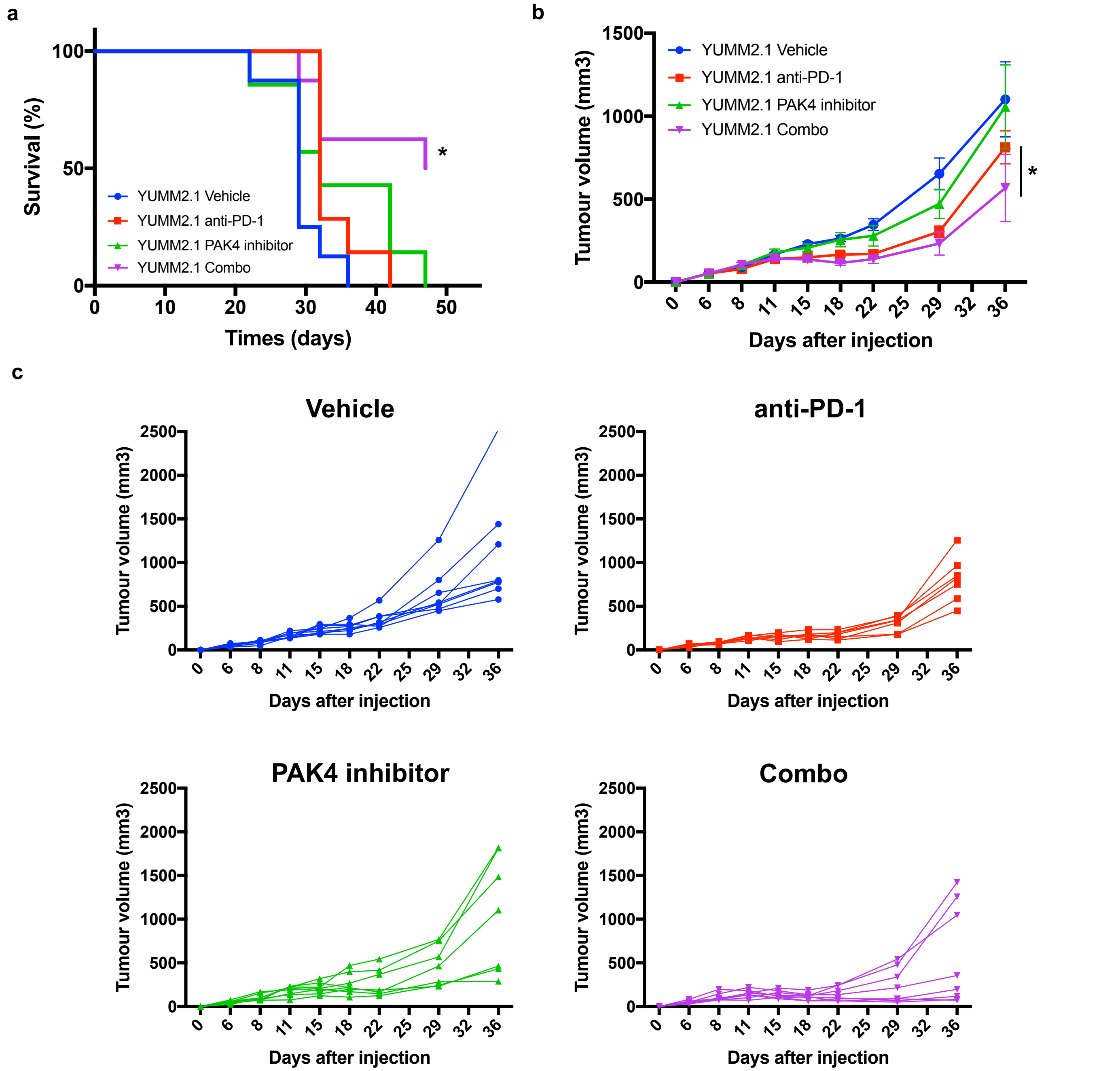

Supplementary Figure 9: PAK4 inhibitor, A0317859, also synergizes with PD-1 blockade in YUMM2.1 melanoma cells. a, Long-term survival for for YUMM2.1 tumors treated with isotype (n = 8, blue), anti-PD-1 (n = 7, red), PAK4 inhibitor (n = 7, green) and combination of PAK4 inhibitor and anti-PD-1 (n = 8, purple). Differences in survival were examine using log-rank (Mantel-Cox) test. Combination of anti-PD-1 with PAK4i significantly increases the survival compared to anti-PD-1 (P = 0.03) and PAK4i (P = 0.03) alone. **b, c,** Growth curves of same YUMM2.1 tumors as in **a**. Pharmacological inhibition of PAK4 kinase activity has a significantly higher anti-tumor activity compared to anti-PD-1 treatment alone (P = 0.04, day 36). Statistical significance and correction for multiple comparisons was calculated using Holm-Sidak method. We also, performed a mixed effect model analysis which resulted in the following slopes (w/SE) for each group: Isotype: 28.9 (2.3), anti-PD-1: 18.3 (2.4), PAK4 inhibitor: 25 (2.4) and combination: 12.4 (2.3) and the following pairwise comparisons were performed: combination vs PAK4 (P < 0.001) and combination vs anti-PD-1 (P = 0.073). *, P < 0.05.
