# Supplementary material for: Remodeling of the Tumor Microenvironment Through PAK4 Inhibition Sensitizes Tumors to Immune Checkpoint Blockade
Source: Cancer Res Commun. 2022 Oct 19;2(10):1214–28. doi: 10.1158/2767-9764.CRC-21-0133 (PMC9799984; doi:10.1158/2767-9764.CRC-21-0133)
Supplement: Supplementary Figure 10 — PAK4 kinase inhibition modulates WNT signalling in vivo. [file crc-21-0133-s10.pdf]

Supplementary Fig. S10

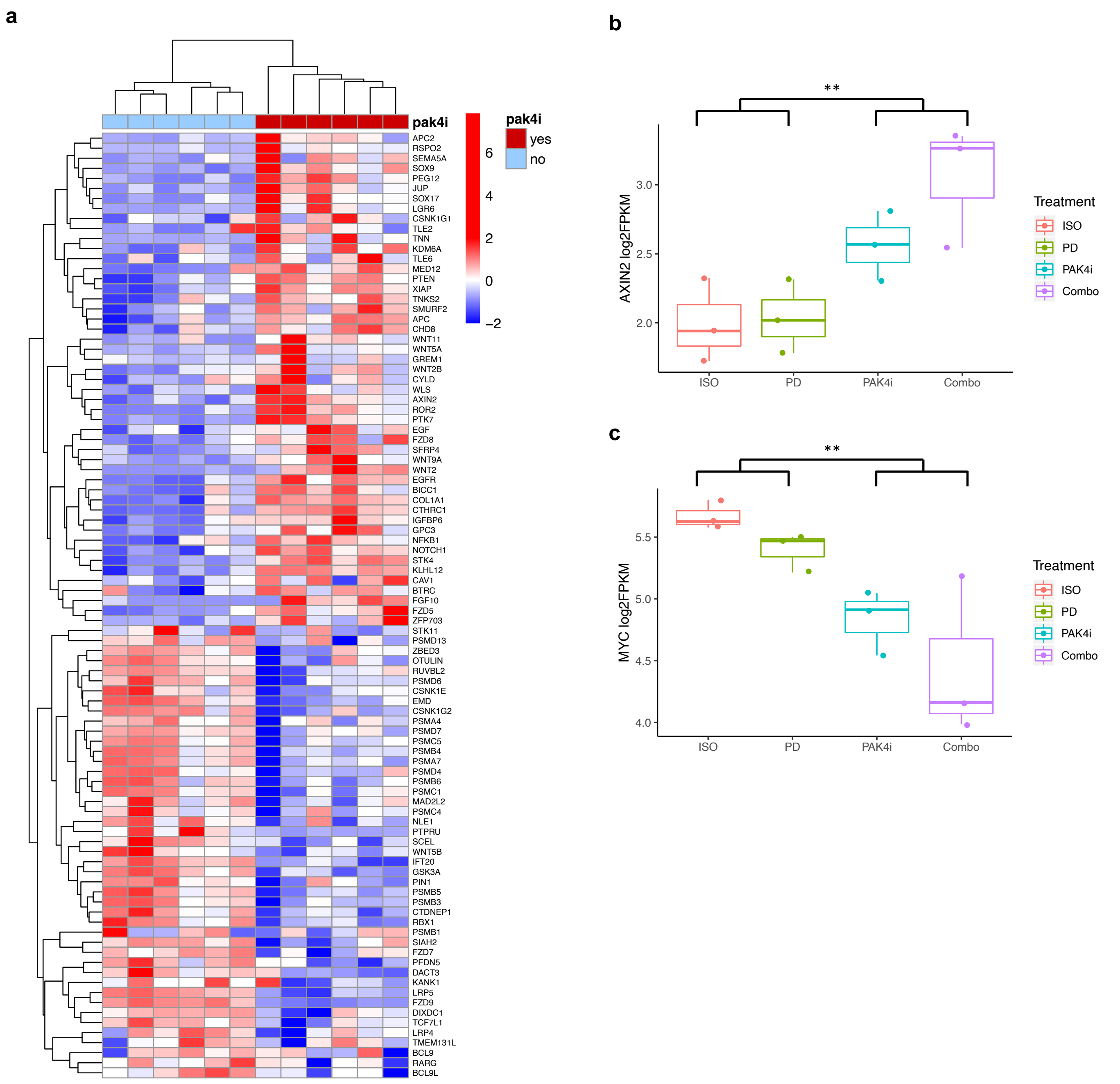

Supplementary Figure 10: PAK4 kinase inhibition modulates WNT signalling *in vivo*. a, Heatmap (row z-scores) of differentially expressed genes between PAK4i treated vs non-treated samples that belong to the gene ontology (GO) canonical WNT signaling pathway. **b,c,** Boxplots showing the log2FPKMs of WNT target genes Axin2 (b) and Myc (c) in each of the four *in vivo* groups. Statistical significance for **b and c** was calculated using a two-tailed un-paired t-test. **P < 0.01
